# Single-cell landscape in mammary epithelium reveals bipotent-like cells associated with breast cancer risk and outcome

**DOI:** 10.1038/s42003-019-0554-8

**Published:** 2019-08-09

**Authors:** Weiyan Chen, Samuel J. Morabito, Kai Kessenbrock, Tariq Enver, Kerstin B. Meyer, Andrew E. Teschendorff

**Affiliations:** 1 CAS Key Laboratory of Computational Biology, CAS-MPG Partner Institute for Computational Biology, Shanghai Institute of Nutrition and Health, Shanghai Institutes for Biological Sciences, University of Chinese Academy of Sciences, Chinese Academy of Sciences, 320 Yue Yang Road, Shanghai, 200031 China; 2 Chao Family Comprehensive Cancer Center, University of California, Irvine 839 Health Science Road, Sprague Hall 114 Irvine, Irvine, CA 92697-3905 USA; 3 UCL Cancer Institute, Paul O’Gorman Building, University College London, 72 Huntley Street, London, WC1E 6BT United Kingdom; 4 Wellcome Sanger Institute, Cambridge, CB10 1SA UK

**Keywords:** Adult stem cells, Statistical methods, Cancer

## Abstract

Adult stem-cells may serve as the cell-of-origin for cancer, yet their unbiased identification in single cell RNA sequencing data is challenging due to the high dropout rate. In the case of breast, the existence of a bipotent stem-like state is also controversial. Here we apply a marker-free algorithm to scRNA-Seq data from the human mammary epithelium, revealing a high-potency cell-state enriched for an independent mammary stem-cell expression module. We validate this stem-like state in independent scRNA-Seq data. Our algorithm further predicts that the stem-like state is bipotent, a prediction we are able to validate using FACS sorted bulk expression data. The bipotent stem-like state correlates with clinical outcome in basal breast cancer and is characterized by overexpression of *YBX1* and *ENO1*, two modulators of basal breast cancer risk. This study illustrates the power of a marker-free computational framework to identify a novel bipotent stem-like state in the mammary epithelium.

## Abstract (web summary)

Chen et al build on a marker-free entropy-based algorithm to assign specific potency states to individual cells using single-cell RNA sequencing data. They identify a putative bipotent stem-cell like state, which associates with basal breast cancer risk and poor clinical outcome.

## Introduction

Single-cell RNA-sequencing (scRNA-Seq) studies are revolutionizing our understanding of cellular development, helping us elucidate the hierarchical organization of cell-types within complex tissues and how this organization may be altered in diseases like cancer^1–14^. An outstanding challenge is how best to identify progenitor or stem-like cells within the large single-cell populations. This task is particularly important for understanding oncogenesis, since the prevailing view is that it is the adult progenitor/stem-like cells that give rise to cancer^15–18^. Specifically, it is believed that inherited molecular alterations, as well as somatic ones that accrue in these cells as a function of age and exposure to risk factors, may eventually predispose these cells to oncogenic transformation.

So far, the most common approach to identify progenitor/stem-like states in scRNA-Seq data, has been to use prior knowledge of specific progenitor or stemness markers, which however may inevitably introduce bias^19–21^. In certain circumstances, this bias can be substantial, specially if knowledge of suitable markers is not available or at best controversial, as is the case for the mammary epithelium^22,23^. Moreover, the high technical dropout rate of scRNA-Seq data means that reliance on well-established markers may not be possible^24^. In this regard, it is worth emphasizing that lineage-trajectory inference algorithms^4,14,25–27^, including recent state-of-the-art ones such as Monocle-3^28,29^, still require specification of a “root-state”, in order to give the trajectories a “temporal” direction, or to define differentiation potency gradients. In the absence of temporal data, the specification of this root state may rely on existing biological knowledge and therefore equally subject to bias. Or the high-dropout rate of scRNA-Seq data may preclude the use of traditional stemness markers to assign this root-state. Another related and key problem is that cell-types are typically inferred as clusters of relatively high cell density in a two-dimensional reduced space, a procedure which does not necessarily allow for the identification of cellular states^19^. Cellular states such as cell-cycle phase or differentiation potency represent additional dimensions of variation, which are generally not well captured or observed by single-cell dimensional reduction and clustering methods. For instance, a single-cell cluster may typically include cells from different cell-cycle stages. Or how to identify novel progenitor or stem-like states within a cell-type may not be possible, using two-dimensional clustering alone, since potency/stemness may be defined by additional latent dimensions.

Here, we show that these outstanding challenges can be overcome with a marker-free system biology approach, called LandSCENT (Landscape of Single Cell Entropy), which builds upon our SCENT framework^30^ to assign each cell, not only to a specific cell-type, but also to a specific potency/entropy state. We stress that the assignment of cells to potency states is achieved without the need for prior knowledge or assumptions, using a potency model that has been extensively validated across many independent scRNA-Seq and bulk RNA-Seq data sets, irrespective of cell lineage, technology, or species^30,31^. LandSCENT combines the inferred cell-types and potency states into a multilayered single-cell landscape, where cell-states are defined by clusters of single cells within a potency state. This allows cells to be placed into specific cellular states, thus allowing novel cellular phenotypes to be identified, for instance novel progenitor or stem-like states within complex epithelial tissues. Importantly, this also allows a natural and unbiased assignment of a root-state, as the one of highest potency, from which lineage trajectories and bifurcation patterns can be subsequently learned using appropriate algorithms such as Diffusion Maps^27,32,33^. We illustrate LandSCENT in the context of the breast epithelium, constructing a combined cell-type and potency landscape at the single-cell level, which, in conjunction with diffusion maps, predicts a novel bipotent progenitor or stem-like cell-state. We provide extensive validation of the bipotent stem-like nature of this state in many orthogonal bulk expression data sets, as well as in scRNA-Seq assays from two different technologies, encompassing altogether data from six different women. We point out that all these results would not have been obtained, had we used competing state-of-the-art clustering or lineage-trajectory inference methods, highlighting the importance of the LandSCENT/SCENT paradigm.

## Results

### Rationale for a marker-free approach to identify stem-like cells

**Fig. 1:**
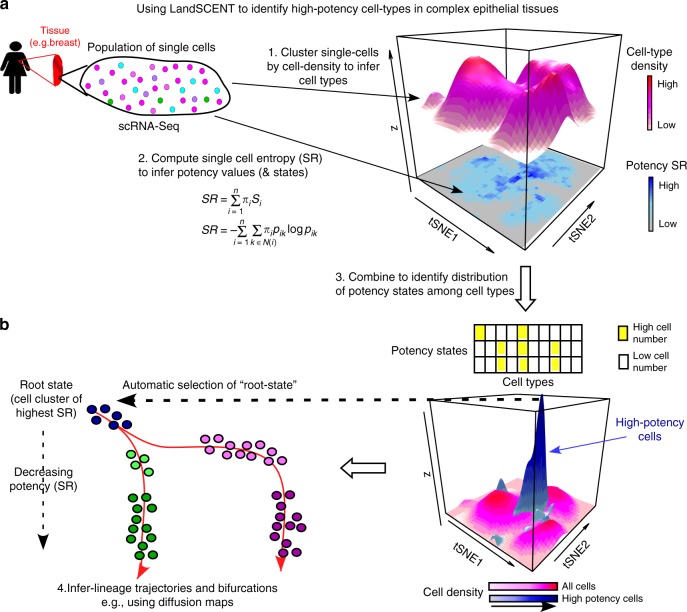
Using LandSCENT to identify novel progenitor or stem-like states in static snapshot scRNA-Seq data. **a** For a given scRNA-Seq dataset profiling thousands of cells in a given tissue type we estimate each single cell’s potency using our Signaling Entropy Rate (SR) measure, in addition to inferring cell-types using ordinary dimensional reduction and clustering (e.g., PCA + tSNE + DBSCAN). This yields two landscapes: a cell-density landscape representing the different cell-type clusters, and a landscape of potency (SR), which we depict here as a heatmap at the bottom. Potency states can be inferred by fitting mixture of Gaussians to the logit-transformed SR values. **b** By counting cells in each potency state and single cluster combination we can identify cell-states, i.e., specific pairs of potency-states and clusters with sufficient representation of cells. In addition, using cell-density elevation maps for highly potent cells can reveal a cell-density landscape very different to that of all cells together, as shown. Finally, the state of highest potency (SR) is identified and assigned as a root-state to infer lineage trajectories using an algorithm such as Diffusion Maps

We reanalyzed scRNA-Seq data from a previous study that used the 10X Genomics Chromium assay to profile over 25,000 mammary epithelial cells from four nulliparous healthy women^34^. We note that due to the high dropout rate of the 10X data, this study had not been able to use the 10X data to confidently identify a stem-like state^34^. We verified that the median dropout rate per cell was over 90% for each of the four women, affecting some of the proposed stemness markers like *ALDH1A1, ZEB1*, and *TCF4*^34,35^ (Supplementary Fig. 1A, B). For instance, for *ZEB1* and *TCF4*, the two stemness markers proposed by Nguyen et al.^34^, the number of cells with a read count larger than 2 in each of the four women was only 1, 0, 0, and 0 for *ZEB1* and only 1, 2, 0, and 1 for *TCF4*, despite thousands of cells having been measured in each woman. Thus, in the absence of stemness marker expression, and to avoid potential biases associated with picking ab initio other markers like *CD44* or *ITGA6*, we decided to apply our marker-free single-cell signaling entropy (SCENT)^30,31^ model, which provides robust estimates of cell potency^14,36,37^. We posited that exploring the distribution of inferred potency values across single-cell clusters may help to identify novel cell-states, including a putative bipotent progenitor or stem-like state. LandSCENT achieves this by combining maps of cell potency and single-cell clusters within a novel “cell-density” visualization framework, which could naturally reveal novel single-cell states (Fig. 1a, the “Methods” section). Importantly, the estimation of cell potency for each single cell allows potency gradients to be naturally inferred, therefore allowing unbiased assignment of “root-states” (i.e., states of highest potency), which can be subsequently used as input for lineage-trajectory inference algorithms (Fig. 1b, the “Methods” section).

### LandSCENT predicts a high-potency state enriched in basal cells

**Fig. 2:**
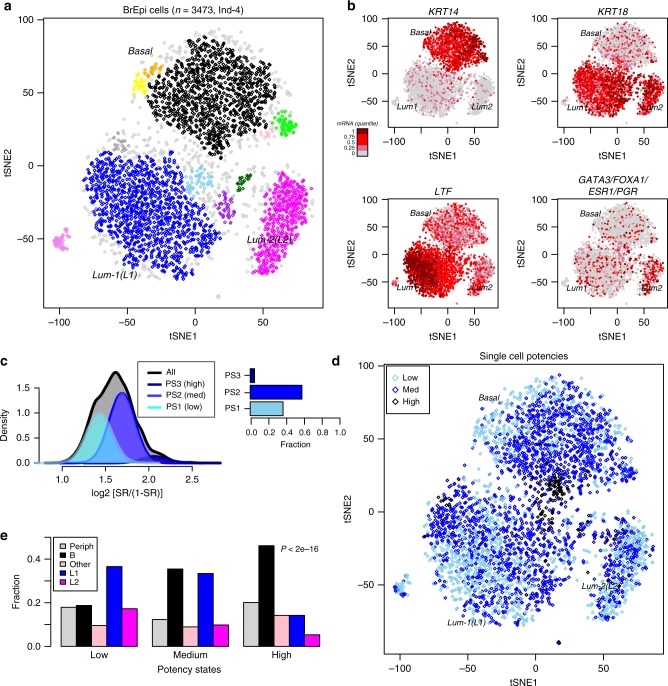
Inferring cell-types and potency states in breast epithelium. **a** t-SNE clustering diagram for single-cells derived from one individual (Ind-4). Single-cell clusters were inferred with DBSCAN and are labeled with different colors. Single-cells that mapped to the periphery of clusters are shown in light-gray color. **b** As A), but now with the single cells labeled by expression levels of *KRT14* (a basal marker), *KRT18* (a luminal marker), *LTF* (lactotransferin), and mean combined expression of *GATA3, FOXA1, ESR1,* and *PGR*, as indicated. Different quantiles of expression levels of each marker are indicated by color with brown indicating high expression and gray low expression. **c** Gaussian mixture model fit to the logit transformed SR values (*x*-axis) from 3473 single cells infers three potency states. The density distributions for all cells (black line) and those for the inferred mixture components (different shades of blue) are shown. The Bayesian Information Criterion (BIC) was used to select the optimal number of potency states, which in this case was found to be 3 (PS1, PS2, PS3). Percentage barplot indicating the fraction of single-cells assigned to each of the three potency states. **d** As A), but now color-labeling single-cells by their inferred potency state (see C)). **e** Percentage barplots displaying the relative distribution of breast epithelial subtypes (as inferred from the clustering using t-SNE + DBSCAN) among inferred potency states (Low, medium, and high). Single cells have been divided up into whether they clustered into the basal compartment (B), into the luminal-1 cluster (L1), the luminal-2 cluster (L2), all other clusters (Other) or whether they were not assigned into any cluster, defining peripheral cells (Periph). *P*-value is from a Kruskal–Wallis test to assess if the distribution of subtypes differs significantly within the high potency state

We observed that only for one of the four women (denoted “Ind-4”) did the top principal component of variation correlate with expression of basal and luminal markers (Supplementary Fig. 2). For the other three women, the top PC correlated with total read count and coverage, accounting for twice as much variance as lower ranked biological components (Supplementary Fig. 2), suggesting that these scRNA-Seq assays were not particularly successful. Thus, we decided to apply LandSCENT to the 3473 single epithelial cells that survived quality control from Ind-4. Performing t-SNE^38^ followed by density-based spatial clustering^39^ revealed three main single-cell clusters (Fig. 2a, the “Methods” section), in line with previous observations^34^, and consistent with known biology: one cluster expressed high levels of *KRT14*, a well-known basal marker, whereas the other two expressed *KRT18*, a well-known luminal marker (Fig. 2b). Consistent with the report of Nguyen et al.^34^, the two luminal clusters were distinguished by expression of lactotransferin (*LTF*) and luminal differentiation markers (*GATA3/FOXA1*), as well as hormone receptors (*ESR1/PGR*) (Fig. 2b), suggesting that the higher *LTF*-expressing cluster represents a more immature (alveolar-like) luminal phenotype. Next, we estimated the differentiation potency of each single cell using our Signaling Entropy Rate (SR) measure (“Methods” section), which revealed the existence of three main potency states (Fig. 2c, the “Methods” section). Of note, using known luminal and basal differentiation markers, we were able to validate potency-state assignments within the basal and luminal clusters separately (Supplementary Notes). We observed that the highest potency state represented a minority population, with approximately only 169 single cells (i.e., 5%) falling into this putative progenitor or stem-like state (Fig. 2c). To explore the biological characteristics of this state, we assessed the distribution of potency states across the three main single-cell clusters, as well as across those cells not assigned to any cluster (“peripheral cells”) (Fig. 2d, e). Cells in the high potency state were found primarily within the basal compartment, but also mapped preferentially to the common peripheral area between the basal and immature luminal clusters, and were therefore also relatively overrepresented among peripheral cells (Fig. 2d, e).

### LandSCENT diffusion map analysis predicts a bipotent state

**Fig. 3:**
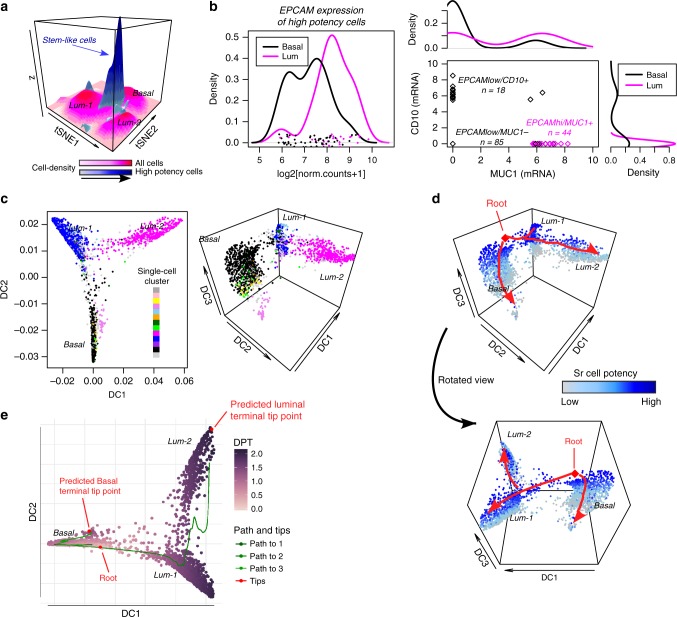
LandSCENT diffusion map analysis predicts a stem-like bipotent state. **a** Surface cell-density map of all single cells (magenta colored surfaces) with the corresponding surface cell-density map of highly potent (PS3) cells superimposed (blueish colored surfaces). The *x* and *y*-coordinates label the t-SNE1 and t-SNE2 axes. The height of the surfaces (*z*) is a measure of cell-density in the *x*–*y* plane and is further indicated by different color tones. The *z*-axis is therefore not a measure of cell potency. **b** Density distribution of EPCAM expression over all high potency cells and stratified according to whether cells fell into the basal or luminal clusters, as indicated. Scatterplot of CD10 versus MUC1 expression for all high potency cells, with corresponding density plots superimposed on the margins. **c** Diffusion map analysis, displaying the distribution of cells along the first two (left panel) and first three (right panel) diffusion components (DC). Cells have been colored according to their original single-cell cluster (see Fig. 2a). **d** Diffusion map of the single cells along the top three diffusion components, but with cells now colored according to their SR potency estimate, as indicated. Bottom view is a rotated version of top panel. The root cell, i.e., the cell of highest potency is indicated in red and putative lineage trajectories displaying the bifurcation into basal and luminal lineages are shown in red. **e** Diffusion Pseudotime (PDT) analysis with the same root state confirming the bifurcation into basal and luminal lineages, with terminally differentiated luminal cells (Lum-2) reached only through an intermediate immature luminal cluster (Lum-1)

To explore the high-potency state in more detail, we first used LandSCENT to create cell-density elevation maps for all cells, and separately also for all highly potent cells, within the two-dimensional t-SNE landscape, which confirmed that the maximum density of the highly potent cells defined a peak within the basal cluster, but with a ridge connecting it to another peak within the immature luminal (L1) cluster (Fig. 3a), suggestive of a bipotent cell population. In line with this, we observed that among all cells categorized into the high potency (PS3) state, those falling within this density peak also exhibited the highest levels of signaling entropy (i.e., cell potency) (Supplementary Fig. 3). To exclude the possibility that these putative bipotent cells may be doublets, we estimated doublet scores for all cells using a novel simulation approach^40^. In line with the expected doublet rate for 10X technology, this analysis revealed that ∼2% of assayed cells are potential doublets (Supplementary Fig. 4A). As expected, most of these mapped to the peripheral area between the major luminal and basal clusters, yet they clearly also did not substantially overlap with the most highly potent cells within the basal and luminal clusters (Supplementary Fig. 4B–D): in fact, 108 of the 169 highly potent cells (i.e., 64%) had zero doublet scores, and only 17 of the 169 highly potent cells, i.e., as few as 10%, attained high doublet scores (Supplementary Fig. 4C), clearly indicating that a substantial majority of the highly potent cells are not doublets. We verified that similar results were obtained had we used another method for estimating doublet scores (Supplementary Fig. 5, the “Methods” section).

Although investigation of specific marker expression is difficult in this high dropout 10X data (Supplementary Fig. 1C), we nevertheless explored the variation in expression of proposed markers for bipotent, luminal-restricted progenitor, and myoepithelial-restricted progenitor cells^41^. Focusing on the highly potent cells, we first observed that although all these cells expressed EPCAM, that those falling within the luminal clusters exhibited higher levels of EPCAM expression compared to those mapping to the basal compartment (Fig. 3b), consistent with the view that luminal-restricted progenitors express higher levels of EPCAM^41^. Next, we plotted the expression of MUC1 versus CD10 for all the highly potent cells, as EPCAMhi/MUC1+ and EPCAMlow/CD10+ cells have been proposed to be luminal-restricted and myoepithelial-restricted progenitors, respectively, whilst EPCAMlow/MUC1- cells are enriched for bipotent progenitors^41^. This scatterplot revealed three substates: an exclusively basal cluster (*n* = 18) expressing high levels of CD10 but MUC1-, a predominantly luminal CD10-/MUC1+ cluster (*n* = 44), and a larger double negative CD10-/MUC1- cluster (*n* = 85) (Fig. 3b). The CD10-/MUC1- cluster was made up 38 basal cells, 11 Lum-1 cells, 4 Lum-2 cells, in addition to 32 peripheral cells, i.e., cells mapping in-between the basal and immature luminal clusters. Thus, these data are highly consistent with the prevailing view that the CD10+ subpopulation correlates with a basal-restricted progenitor subtype, that the MUC1+ subpopulation associates with a luminal restricted progenitor subtype, and that the MUC1-/CD10- cluster contains a bipotent subtype.

In order to substantiate the above findings, we next applied Diffusion Maps, a powerful tool for inferring bifurcation points and lineage trajectories in scRNA-Seq data^27,32^. We observed that while diffusion components 1 and 2 correlated strongly with the three main clusters (basal, Lum-1, and Lum-2) (Fig. 3c), that diffusion component 3 was highly correlated with our SR cell potency measure (Fig. 3d). Defining as root state the cell of highest SR (i.e., potency), this cell mapped to the periphery of the basal cluster and the resulting diffusion map naturally predicts a bifurcation from this root state into marginally lower but still high-potency basal and luminal states (Fig. 3d). Differentiated basal and luminal clusters emerge from these restricted progenitor states along their respective basal and luminal lineages, as required (Fig. 3d). Confirming this, diffusion pseudotime (DPT) analysis predicted two major terminal tip-points, one in the basal cluster and another in the mature luminal-2 state, with no direct transition between the basal and luminal-2 clusters (Fig. 3e), i.e., DPT analysis correctly predicts that the mature luminal-2 state is only reached after passing through the immature luminal-1 cluster, consistent with it containing the luminal progenitor population.

### Validation of the single-cell stem-like state

**Fig. 4:**
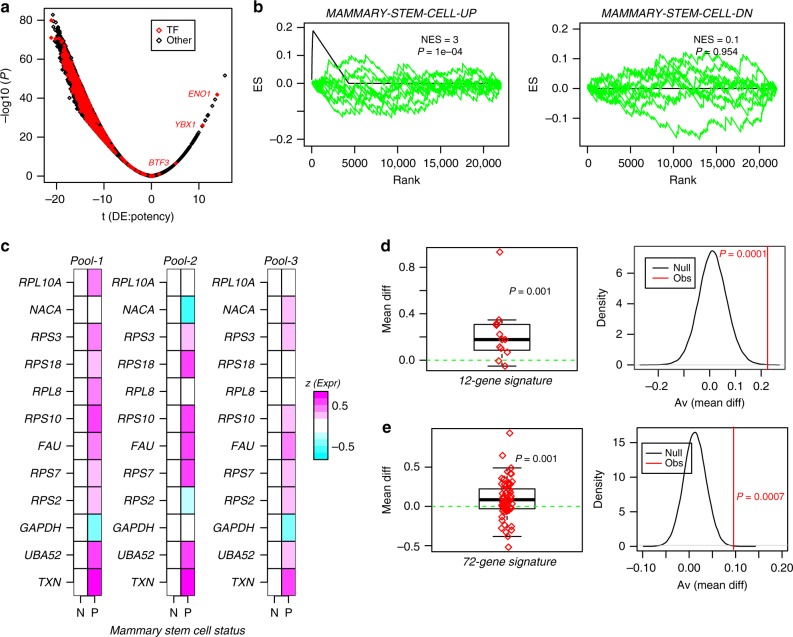
Validation of single-cell stem-like signature with GSEA and mammosphere-derived data. **a** Volcano plot of differential expression associated with potency, with *x*-axis labeling the *t*-statistic and *y*-axis labeling the statistical significance. Horizontal bar denotes the Bonferroni threshold, and red points indicate transcription factors (TFs). **b** Plots of the Enrichment Score (ES, *y*-axis) from rank-based GSEA against rank index position (*x*-axis) for genes ranked according to their positive correlation with potency as assessed using the scRNA-Seq data (black line), and for two different biological terms from the MSigDB dataset: genes upregulated and downregulated in mammary stem cells (Pece et al.). Green curves describe dependence of the ES score on rank position after Monte-Carlo randomization of the gene-ranking, for ten different Monte-Carlo runs. The Normalized Enrichment Score defined by the ratio of the observed maximum ES score to the mean of the maximum over 1000 Monte-Carlo runs is given, as well as the associated *P*-value derived by approximating the max ES scores over the 1000 Monte-Carlo runs as a Gaussian. **c** Normalized relative expression heatmaps for the 12 genes upregulated in the putative stem-like single-cells and which overlap with a mammary stem-cell signature, in three separate pools of FACS sorted quiescent mammary stem-cells (P) and their derived proliferative non-stem like progeny (N). **d** Average expression difference between the P and N cells, averaged over the three separate pools. *P*-value is from a one-tailed Wilcoxon rank sum test. Monte-Carlo randomization analysis, where in each of 100,000 random selections of 12 genes, the average difference over the three pools is computed (greycurve) and compared to the observed average difference (red, panel-B). Monte-Carlo *P*-value is given. **e** As D), but for the 72-gene bipotent signature

If the bipotent cell cluster identified by LandSCENT is stem-like, the expectation would be that these cells may be transcriptionally similar to previously characterized mammary stem cells. To explore this, we performed differential expression analysis between high and low potent cells. The great majority of genes were downregulated in the more potent cells, with only 72 exhibiting overexpression (Bonferroni adjusted *P* < 0.05, Fig. 4a, Supplementary Table 1). Remarkably, performing rank-based GSEA^42^ on the 72 overexpressed genes revealed strong enrichment for genes upregulated in mammary stem-cells (Fig. 4b). In particular, we observed a relatively strong enrichment (12 gene overlap, OR = 39, BH-adjusted Fisher-test *P* < 1e−10) with a previously characterized mammary stem-cell signature^43^. Of note, among the 12 overlapping genes, 9 (*RPS2, RPS7, RPS10, RPL8, RPS18, RPS3, RPL10A*) were ribosomal proteins or ubiquitin ribosomal fusion proteins (*UBA2 and FAU)*, consistent with recent findings that expression of ribosomal proteins may be a universal marker of stemness and potency^30,44^ (Supplementary Fig. 6). We stress that the higher mRNA expression levels of ribosomal genes with increased cell potency is also observed in bulk samples^30,36^, thus excluding the observed association as an artifact of single-cell analysis. Among the other three genes, we observed *NACA*, a protein that associates with the upregulated transcription factor *BTF3*, and *TXN* (thioredoxin), a protein involved in the response to intracellular nitric oxide.

To confirm the results of the GSEA, we obtained and normalized mRNA expression data from^43^, consisting of FACS sorted pools representing quiescent mammary stem-cells and transit-amplifying progenitors, as derived from mammosphere-growing assays (“Methods” section). Confirming the association with stemness, the 12 overlapping genes exhibited increased expression in three separate pools of quiescent mammary stem-cells compared to their derived transit-amplifying progenitors (Fig. 4c, d, Wilcox test *P* = 0.001, the “Methods” section), a result which remained significant compared to randomly selected genes (Fig. 4d, Monte Carlo *P* = 0.0001). Results remained significant had we used all 72 genes (63 genes had representation on the Affymetrix platform used in Pece et al.^43^) from the upregulated stem-like signature (Fig. 4e, Supplementary Fig. 7). Although this validation uses data generated in vitro, and therefore ignores in vivo effects, the data nevertheless support the view that the cells deemed to be stem-like according to our LandSCENT algorithm, are indeed related to mammary stemness. Of note, the identification of the stem-like state was not possible using other state-of-the-art lineage-inference trajectory algorithms such as e.g., Monocle-2^28^ (Supplementary Notes).

### Validation in independent 10X and Fluidigm C1 data

**Fig. 5:**
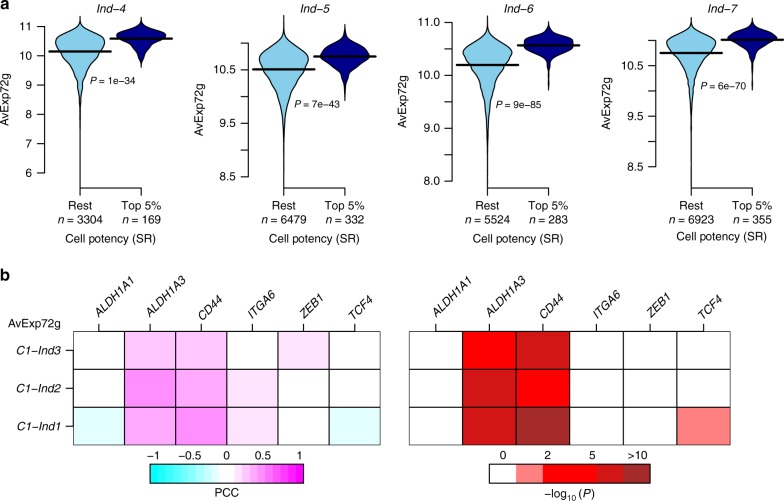
Validation of single-cell stem-like signature in independent 10X and Fluidigm C1 data. **a** Violin plots comparing the average expression over the 72 upregulated genes in the single-cell stem-like signature between the top-5% of most highly potent cells (as derived from our cell potency SR measure) and the rest of cells, for each of the four women for which 10X scRNA-Seq was generated. *P*-values are derived from a one-tailed Wilcoxon rank sum test. **b** Heatmap of Pearson correlation coefficients (PCC) between the average expression of all 72 genes (AvExp72g) making up the single-cell stem-like signature with the expression of six stemness markers (*ALDH1A1, ALDH1A3, CD44, ITGA6, ZEB1*, and *TCF4*), as indicated. The correlations were computed in the Fluidigm C1 scRNA-Seq dataset, separately for three different individuals (*n* = 198 cells for Ind-1, *n* = 195 cells for Ind-2, and *n* = 322 for Ind-3). Right panel heatmap displays the corresponding –log_10_ [*P*-values]

While the quality of the 10X scRNA-Seq assay from the other three women is questionable (Supplementary Fig. 2), we nevertheless aimed to further validate the single-cell stem-like transcriptomic signature in these data. We reasoned that the average expression of the identified 72 upregulated genes should be a stemness marker in the 10X data from these three women. Confirming this, for each woman we observed a significant increased expression of these 72 genes in the single cells deemed to be of highest potency according to our highly validated SR measure (Fig. 5a, Wilcox test *P* < 1e−30).

As a further validation, we would expect the identified stem-like cells to preferentially overexpress previously characterized stemness markers. Despite the high dropout rate of the 10X data (Supplementary Fig. 1), we nevertheless first assessed correlations between the 72 upregulated genes and a panel of 6 stemness markers (*ALDH1A1, ALDH1A3, CD44, ITGA6, ZEB1,* and *TCF4*)^34,35^ in the 10X data, finding small but significant positive correlations for *ALDH1A3, CD44,* and *ITGA6* (Supplementary Fig. 8, Fisher Z, *P* < 1e−5). We further tested for correlations between our upregulated signature genes and expression of the stemness markers in three independent higher-coverage scRNA-Seq datasets from the mammary epithelium generated with the Fluidigm C1 platform^34^ (“Methods” section). We observed a statistically significant correlation with *ALDH1A3* and *CD44* expression (Fig. 5b, Fisher Z, *P* < 1e−10). Thus, while the stem-like state identified in the 10X data from Ind-4 is clearly not identifiable via single stemness marker expression, we observed partial but significant correlations with *ALDH1A3* and *CD44* in both 10X and Fluidigm C1 data.

### Single-cell stem-like signature is increased in luminal progenitors

**Fig. 6:**
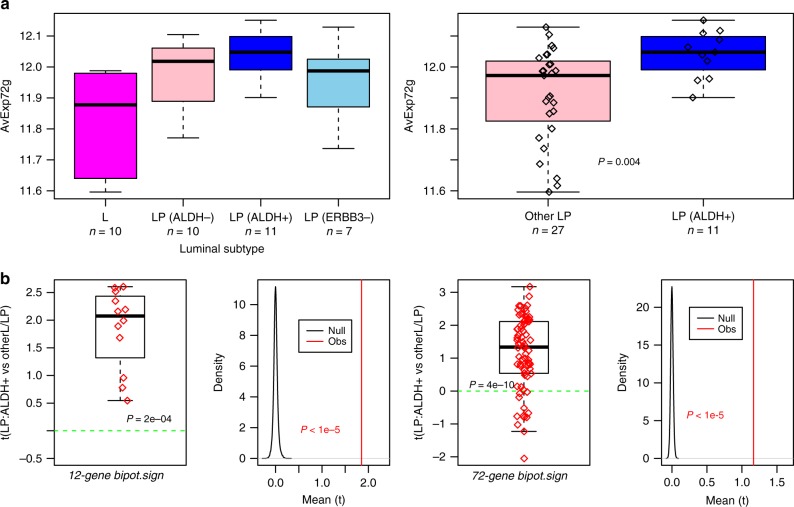
Bipotent single-cell expression signature is elevated in ALDH+ luminal progenitors. **a** Boxplots of the average expression over the 72 upregulated genes in the bipotent stem-like single cell signature, against luminal subtype as defined by FACS-sorting (*x*-axis): L = differentiated non-clonogenic luminal, LP(ALDH-) = ALDH- luminal progenitor, LP(ALDH+) = ALDH + luminal progenitor. LP(ERBB3-) = ERBB3- and ALDH- luminal progenitor. As right, but comparing the ALDH + subtype to the rest. *P*-value is from a one- tailed Wilcox test comparing LP(ALDH + ) to all others. **b** Boxplot of *t*-statistics of differential expression between the EPCAM + /ITGA6+/ALDH+ cells (*n* = 11) and all other luminal and putative luminal progenitors (*n* = 27) from the FACS bulk data study of Shehata et al., displaying these statistics for 12 genes upregulated in the bipotent single-cell cluster and which also map to the mammary stem-cell signature of Pece et al. Monte-Carlo derived null distribution of the mean *t*-statistic for 12 randomly selected genes (100,000 runs). As left panels, but now for all the 72 genes defining the upregulated bipotent signature

Having validated the stem-like nature of the highly potent cell cluster, we next asked if the transcriptome of these cells may also mark luminal progenitors (LPs). This is reasonable, because although the highly potent cells were mostly enriched in the basal cluster, a considerable number did map to the more immature luminal cluster, occupying a topologically central position close to those in the basal cluster (Fig. 3a). To test our hypothesis, we analyzed bulk expression data from four FACS sorted cell populations, three representing putative LP subclasses and one representing differentiated luminal cells^45^. We observed that the average expression of the 72 upregulated genes was highest for the EpCAM + /ITGA6 + /ALDH + luminal progenitor population (Fig. 6a, Wilcox test *P* = 0.004), consistent with the view that it is the ALDH + cells that are most likely to represent LPs^45^. Studying the individual genes in the 12-gene and 72-gene signatures, revealed that the great majority were overexpressed in the EpCAM + /ITGA6 + /ALDH + population compared to all other luminal/LP populations, a result which was highly significant as assessed using 100,000 Monte-Carlo randomizations (Fig. 6b, *P* < 1e−5). These data further support the view that the identified stem-like state may be bipotent, as it shares similarity with both basal and luminal progenitors.

### Bipotent-like cells are marked by *YBX1* and *ENO1* overexpression

As noted earlier, the great majority of genes were downregulated in the stem-like cell cluster, with only 72 exhibiting overexpression. Correspondingly, among the 1369 transcription factors, 582 exhibited differential expression (Bonferroni adjusted *P* < 0.05) with only 3 TFs (*ENO1, YBX1,* and *BTF3*) exhibiting higher expression in the more potent cells (Fig. 4a). Remarkably, *YBX1* and *ENO1* are two transcription factors whose targets are highly enriched for breast cancer GWAS eQTLs^46^, thus implicating them in breast cancer risk. In addition, siRNA against *YBX1* in a normal ER- cell-line (MCF10A) resulted in significantly reduced cell-confluence and growth, even when compared to other breast cancer risk TFs^46^. We confirmed that the associations of *YBX1* and *ENO1* expression with potency remained after adjustment for cell-cycle phase (Supplementary Fig. 9, the “Methods” section), and that their expression correlated with cell potency in the 10X scRNA-Seq data from each of the four women (Supplementary Fig. 10). We note that the correlation of *YBX1* expression with potency was particularly evident in the luminal compartment (Supplementary Fig. 11). Moreover, *YBX1* expression was also higher in the more immature luminal alveolar-like phenotype, in line with the fact that these alveolar luminal cells should be more enriched for progenitors, and that YBX1 expression was also highest in the FACS-sorted ALDH+ luminal progenitor population (Supplementary Fig. 12).

Of note, both *YBX1* and *ENO1* also exhibited significant positive correlations with the *ALDH1A3* and *CD44* stemness markers in the Fluidigm C1 data (Supplementary Fig. 13, Fisher Z, *P* < 1e−10), but were not upregulated in the quiescent mammary stem cells compared to the transit-amplifying progenitor cells (Supplementary Fig. 7), suggesting that *YBX1* and *ENO1* expression may be associated with an amplifying (bipotent) progenitor state.

### Bipotent signature marks basal breast cancer and poor clinical outcome

**Fig. 7:**
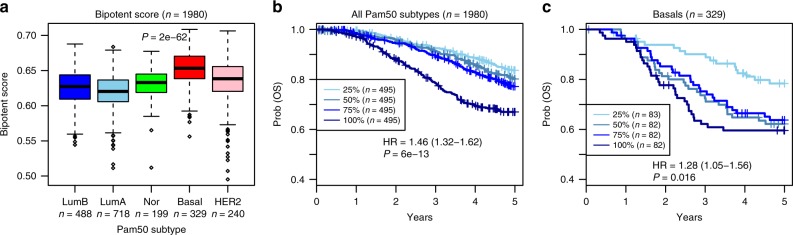
Bipotent single-cell expression signature correlates with clinical outcome within basal subtype. **a** Boxplots of the bipotency score as estimated using the 144-gene bipotency signature in the METABRIC cohort across the PAM50 intrinsic subtypes. *P*-value is from a Wilcox test comparing basals to all others. Number of samples in each subtype are given. **b** Kaplan–Meier overall survival curves for all 1980 METABRIC samples, stratified by quartiles of the bipotency score, and with survival data censored at 5 years after diagnosis. Hazard Ratio (HR), 95%CI and *P*-value are from a Cox proportional hazards regression. **c** As **b**, but now focusing only on the 328 basal samples

Given that our stem-like signature was derived from single cells and is therefore free from the confounding effect of cell-type heterogeneity, we decided to test it in primary breast cancer tissue. Since the stem-like state was enriched within the basal compartment, we hypothesized that the signature may mark basal breast cancer and be prognostic within this subtype. We confirmed the association with basal breast cancer using 2000 primary breast cancers profiled as part of the METABRIC study^47^ (Supplementary Fig. 14). The average expression over the 72 genes was also associated with clinical outcome, although only marginally so in the basal subtype (Supplementary Fig. 15). In order to construct a single-cell derived stemness score, we also considered an expanded 144-gene expression signature which, besides the 72 upregulated genes, included the 72 most significantly downregulated genes within the high potency single-cell cluster (Supplementary Table 1). This strategy allowed us to compute a Pearson correlation between the 144-gene signed signature and the expression profile of each METABRIC sample, which should yield a more robust “stemness/bipotency score” (“Methods” section). This score was also significantly higher in the basal subtype (Wilcox test *P* < 1e−50, Fig. 7a), and correlated with poor clinical outcome (HR = 1.46 (95%CI: 1.32–1.62), *P* = 6e−13, Fig. 7b), which remained significant in a multivariate analysis adjusted for ER-status, grade, age, stage, and tumor size (HR = 1.26 (95%CI: 1.10–1.43), *P* = 0.0006, Supplementary Table 2). Importantly, the association with overall survival remained significant within the basal subtype (HR = 1.28 (95% CI: 1.05–1.56), *P* = 0.02, Fig. 7c) even when adjusted for age, stage, and tumor size (HR = 1.30 (95%CI: 1.02–1.66), *P* *=* 0.03, Supplementary Table 3). The difference in the 3-year overall survival rate between the lowest and highest quartiles was substantial: while those with the lowest stemness score exhibited a 90% 3-year survival rate, those in the highest quartile showed a 30% reduction (Fig. 7c).

## Discussion

Here we have demonstrated “proof-of-concept” that our signaling entropy based cell potency measure can identify rare subpopulations representing novel progenitor or stem-like cells. Indeed, application to almost 4000 single cells from the mammary epithelium identified a minor (<5%) high potency subpopulation, which we argue likely represents a mammary bipotent progenitor or stem-like state. These high-potency cells were not randomly distributed: they were over-represented within the basal compartment, but also mapped preferentially to the periphery of the basal and immature alveolar luminal clusters, with a smaller fraction of marginally lower potency also being exclusive to this luminal cluster. Using a novel visualization technique based on generating and comparing cell-density surface maps for all inferred potency states, confirmed that cells in the high-potency state clustered most strongly at the periphery of the basal cluster, with others defining a distinctive bi-modal ridge between the basal and alveolar luminal clusters. Of note, cells defining the peak of maximum cellular density were also the ones attaining the highest potency values. Without having to invoke any prior assumptions, this topologically central position predicts that these highly potent cells may represent a bipotent stem-like state that gives rise not only to basal cells but also to luminal progenitors, in direct analogy with the topologically central positions observed for e.g., hematopoietic stem cells in the hematopoietic system^48^.

Many analyses substantiate this view. First, using only high-potency cells, a scatterplot of expression of CD10 and MUC1, two markers that have been proposed to differentiate bipotent progenitors from basal-restricted and luminal-restricted progenitors^41^, revealed three states: a CD10+/MUC1- population composed only of basal cells, a CD10-/MUC1+ population composed almost exclusively of immature luminal cells, and a double negative CD10-/MUC1- population which was composed mainly of basal cells, but which also included a number of peripheral “ridge-defining” cells as well as a few immature luminal cells. Thus, consistent with previous literature^41^, the CD10+/MUC1- and CD10-/MUC1+ cells likely represent basal-restricted and luminal-restricted progenitor populations, respectively, with the basal and peripheral CD10-/MUC1- cells defining a bipotent-like state. Second, we used our entropy potency measure to define a natural root-state as the cell attaining the highest potency, from which a diffusion map process was then inferred. This predicted a bifurcation, with one lineage giving rise to basal-restricted progenitors and fully differentiated basal cells, and with the other giving rise to luminal-restricted progenitors and differentiated luminal cells. Third, we found that among the top overexpressing genes in the bipotent stem-like state there was strong enrichment for genes that mark quiescent mammary stem cells^43^ and stemness generally^30,44^. We stress that this validation of the single-cell stem-like state was obtained in bulk mRNA expression data comparing quiescent mammary stem-cells to transit-amplifying progenitors, which therefore strongly reinforces the validity of our potency assignments. Fourth, the stem-like single cell signature, which was derived from the 10X scRNA-Seq assay from one woman, also exhibited variability in the 10X scRNA-Seq assays from another three women, in each case correlating with our highly validated potency measure. Fifth, we found that our stem-like single-cell signature also correlated significantly with the expression of *ALDH1A3* and *CD44*, two well-known putative mammary stem-cell markers in independent higher coverage C1 Fluidigm data from another three women. We stress that although significant correlations with these two markers were also observed in the 10X data, that these correlations were relatively weak and only significant due to the larger number of cells. This is important because we note that using *ALDH1A3* or *CD44* expression itself did not allow identification of the novel stem-like state, even if used in conjunction with a state-of-the-art tool like Monocle-2. Sixth, the single-cell expression signature characterizing this stem-like state was also found to be elevated in FACS sorted ALDH+ luminal progenitor cells compared to differentiated luminal and other less differentiated luminal subtypes. This suggests that the signature is not only marking basal progenitors but also luminal progenitors, further supporting a bipotent interpretation.

Of note, a recent scRNA-Seq study performed in the mouse mammary gland which also used diffusion maps^49^, reached the conclusion that basal and luminal lineages were separate without evidence of a bifurcation, therefore questioning the existence of a bipotent state. Interestingly, this is in line with a recent neutral lineage study in mice^50^, which did not find evidence for bipotent cells in the mammary gland. However, if the bipotent cells are in a highly quiescent state, they may not have been found in such lineage tracing studies^50^. Moreover, a likely explanation for the discrepancy with the mouse scRNA-Seq study is the fact that this previous study did not use an independent potency measure to define a reliable root state. Indeed, reliance on stemness or progenitor marker expression alone to define such a root state does not allow reliable identification of stem-like cells in high dropout rate scRNA-Seq data, as evidenced here but also in this previous study. It is clear that the prediction or not of specific bifurcation points using diffusion maps will depend critically on the identification of a reliable root state, specially since cells transiting between bipotent and lineage-restricted progenitor states are sparse. Thus, it will be necessary to profile even larger numbers of cells and at higher read-depth (average read depth of the 10X data considered here was 60,000 reads per cell) to conclusively address this question. Higher-read depth would allow full characterization of the transcriptome of this bipotent stem-like state, which may in turn help pinpoint specific surface markers.

The putative bipotent state as revealed by LandSCENT may have important implications for basal breast cancer. It is indeed striking that of the three TFs overexpressed in the stem-like state, two (*YBX1* and *ENO1*) have been implicated in basal breast cancer risk^46^. Specifically, it has been observed that genes within the *YBX1* and *ENO1* regulons are strongly enriched for GWAS breast cancer eQTLs^46^. The third TF (*BTF3*) has been shown to be necessary for proliferation and EMT in gastric cancer^51^. *YBX1* merits further study as it has been shown to play a key role in maintaining the self-renewal and proliferative capacity of basal cells^46^. There is also substantial evidence demonstrating that *YBX1* transforms mammary epithelial cells, via binding to the *BMI1* promoter and chromatin remodeling, leading to basal breast cancer^52^. In line with this, *YBX1* is also more highly expressed in basal breast cancer compared to all other breast cancer subtypes (Supplementary Fig. 14). Interestingly, *YBX1* and the associated stem-like signature was also highly expressed in luminal progenitors, which is important because a subset of basal breast cancers, notably BRCA1 mutant ones, are thought to arise from misprogrammed luminal progenitors^45,53^. Indeed, the single-cell landscape inferred with LandSCENT underscores the similarity of the highly potent cells within the basal compartment with those in the immature luminal cluster, strongly suggesting that the cell of origin for basal breast cancer may well be a bipotent-like cell that shares an expression profile similar to that of luminal progenitors. *YBX1* has also been shown to interact with *ESR1*, and via *FGFR2* signaling may contribute to tamoxifen resistance^54^. Interestingly, although the majority of the 72 upregulated genes were also overexpressed in the quiescent mammary stem-cells derived from mammosphere-growing assays, both *YBX1* and *ENO1* were not overexpressed relative to the transit-amplying progenitors, suggesting that they may not be stemness markers per-se, but markers of a bipotent early progenitor state. Beyond *YBX1*, we characterized the putative bipotent cells in terms of a 144-gene “bipotent” expression signature, which clearly marked basal breast cancer, and which also correlated with poor overall survival within the basal subtype independently of standard prognostic factors, all consistent with it defining a “poor outcome stemness signature”. While poor outcome stemness signatures derived from bulk data have been widely reported in breast cancer^55–58^, this study presents a prognostic stemness signature derived from single cells and therefore free from the confounding effects of cell-type heterogeneity. Thus, the observation that the single-cell stem-like signature correlates with clinical outcome in basal breast cancer, whilst also including a TF that is oncogenic for basal breast cancer and which has also been implicated in basal breast cancer risk is in our opinion an important finding. Indeed, there is growing evidence that molecular alterations (both inherited and somatic) affecting the adult stem/progenitor cell pool of a tissue is a main risk factor for epithelial cancer development^16–18,59,60^. Thus, we speculate that it is the genetic and epigenetic alterations that accumulate within the bipotent progenitor cell pool identified here, which may confer the risk of breast cancer, especially basal breast cancer.

In summary, we have here showcased the application of an unbiased marker-free computational approach for estimating cell potency, and which, in an application to the human mammary epithelium, has identified a novel putative bipotent stem-like state, with the transcriptome of these cells exhibiting associations with basal breast cancer risk and outcome. Our LandSCENT algorithm and findings may serve as a general paradigm for analogous scRNA-Seq studies in other tissue types, including those performed on cancer tissue which aim to identify putative cancer stem-cells^5,8,30^.

## Methods

### Single-cell data and preprocessing

*10X Genomics set:* The main scRNA-Seq data analyzed in this work derives from the study of Nguyen et al.^34^, who used the 10X Genomics Chromium platform to sequence a total of 24,646 cells from reduction mammoplastic specimens from four separate nulliparous women (Ind4–7), at an average read-depth of 60,000 reads per cell. Mapped read count data from the four individuals was downloaded from GEO (GSE113197), and further normalized as follows: for each cell we counted the number of expressed genes (“coverage per cell”), and for each gene we also counted the number of times it was expressed across all single cells (“coverage per gene”). For each cell, we also computed the total read count mapping to mitochondrial genes, which revealed low cell coverage for those cells having a high proportion of mitochondrial gene read counts. Based on this, we selected all cells expressing at least 1000 genes and with the proportion of mitochondrial read counts <0.05, leaving a total of 23,369 cells. Mitochondrial genes were removed and the total read count per cell *c* recomputed (TRC_*c*_). Denoting the maximum of *TRC*_*c*_ by *maxC*, and the read count matrix by *RCM*, the latter was normalized with the following transformation: LSC_*gc*_ = *log*_2_(RCM_gc_*maxC/TRC_*c*_ + 1.1). Finally, we only use Entrez gene ID annotated genes, which resulted in a log-normalized single cells matrix of dimension 22,049 genes and 23,369 cells (3473 for Ind-4, 6811 for Ind-5, 5807 for Ind-6, and 7278 for Ind-7).

*Fluidigm C1 set:* In addition, we also analysed the corresponding Fluidigm C1 scRNA-Seq set, also from Nguyen et al.^34^. We downloaded the FPKM-valued matrix of 33,694 features and 815 cells encompassing cells from three different women. We selected cells expressing at least a 1000 genes and with a mitochondrial proportion less than 0.3, leaving a matrix of 33,681 features and 715 cells. The FPKM matrix was log_2_-normalized with a pseudocount of 1. We only kept genes mapping to an entrez gene ID, which resulted in a normalized expression matrix over 22,049 genes and 715 cells. The number of cells for the three individuals were 198 (Ind-1), 195 (Ind-2) and 322 (Ind-3).

### The LandSCENT algorithm

LandSCENT is a direct extension of the SCENT algorithm. There are four steps to the LandSCENT algorithm: (1) Inference of potency states: estimation of the differentiation potency of single cells via computation of the signaling entropy rate (SR) and subsequent inference of the potency state distribution across the single cell population. (2) Inference of cell-types: we perform t-SNE^38^ followed by density-based spatial clustering (dbscan)^39^ on a suitably dimensionally reduced *LSC* matrix. (3) Identification of cell-states, i.e., potency state single-cell cluster pairs that contain a minimum number of cells^30^, and construction of cell-density landscapes for each potency-state. (4) Identification of a root-state, i.e., the cell state of highest entropy/potency (SR), and subsequent application of Diffusion Maps^27,32^ to infer bifurcations and lineage trajectories. We note that step-1 is the exact same procedure as used in our original SCENT algorithm^30^.

Step-1 Inference of potency states: We estimate differentiation potency of each single cell by computing the signaling entropy, as described previously^31,61^. Briefly, the normalized genome-wide gene expression profile of a sample (this can be a single cell or a bulk sample), which provides the biological context, is used to assign weights to the edges of a highly curated protein–protein interaction (PPI) network. The construction of the PPI network itself is described in detail elsewhere^31^, and is obtained by integrating various interaction databases which form part of Pathway Commons (www.pathwaycommons.org)^62^. The PPI network as used here is available from https://github.com/ChenWeiyan/LandSCENT/tree/master/data under filename net13Jun12.m.RData. The weight of an edge between protein *i* and protein *j*, denoted by *w*_*ij*_, is assumed to be proportional to the normalized expression levels of the coding genes in the cell, i.e., we assume that *w*_*ij*_*~x*_*i*_*x*_*j*_, and we interpret these weights (if normalized) as interaction probabilities. Thus, in a sample with high expression of *i* and *j*, the two proteins are more likely to interact than in a sample with low or absent expression of *i* and/or *j*. Normalizing the weights results in a random walk defined by a stochastic matrix, *P*, over the network, with entries

$$p_{ij} = \frac{{x_j}}{{\mathop {\sum }\nolimits_{k \in N(i)} x_k}} = \frac{{x_j}}{{(Ax)_i}}$$

where *N(i)* denotes the neighbors of protein *i*, and where *A* is the adjacency matrix of the PPI network (*A*_*ij*_ *=* *1* if *i* and *j* are connected, 0 otherwise, and with *A*_*ii*_ *=* 0). The signaling entropy is then defined as the entropy rate (denoted *Sr*) over the weighted network, i.e.,

$$Sr\left( {\vec x} \right) = - \mathop {\sum }\limits_{i = 1}^n \pi _i\mathop {\sum }\limits_{j \in N(i)} p_{ij}\log p_{ij}$$

where *π* is the invariant measure, satisfying *πP* *=* *π* and the normalization constraint *π*^*T*^**1** = 1. The invariant measure, also known as steady-state probability, represents the relative probability of finding the random walker at a given node in the network (under steady state conditions i.e., long after the walk is initiated). Nodes with high values thus represent nodes that are particularly influential in distributing signaling flux in the network. In the steady-state we can assume detailed balance (conservation of signaling flux, i.e., *π*_*i*_*p*_*ij* =_ *π*_*j*_*p*_*ji*_), and it can be shown^61^ that *π*_*i*_ = *x*_*i*_(*Ax*)_*i*_/(*x*^*T*^*Ax)*. Given a fixed adjacency matrix *A* (i.e., fixing the topology), it can also be shown^61^ that the maximum possible *Sr* among all compatible stochastic matrices *P*, is the one with $P = \frac{1}{\gamma }v^{ - 1} \otimes A \otimes v$ where ⊗ denotes product of matrix entries and where *v* is the dominant eigenvector of *A*, i.e., *Av* = *λv* with *λ* the largest eigenvalue of *A*. We denote this maximum entropy rate by *maxSr*, and define the normalized entropy rate (with range of values between 0 and 1) as

$$SR\left( {\vec x} \right) = \frac{{Sr(\vec x)}}{{maxSr}}$$

Since SR is bounded between 0 and 1, we next transform the SR value of each single cell into their logit-scale value, i.e., *y*(SR) = *log*_2_*(*SR*/*(1−SR)). Subsequently, we fit a mixture of Gaussians to the *y(*SR*)* values of the whole cell population, and use the Bayesian Information Criterion (as implemented in the *mclust* R-package)^63^ to estimate the optimal number *K* of potency states, as well as the state-membership probabilities of each individual cell. Thus, for each single cell, this results in its assignment to a specific potency state.

Step-2 Inference of cell-types*:* Cell-types are inferred as clusters using cell-density in the two-dimensional t-SNE space as the main criterion. Preliminary dimensional reduction is achieved by first selecting genes with a mean average expression larger than 1, and also a standard deviation larger than 1. These thresholds were chosen after inspection of the mean-variance plot, and in the case of Ind-4 this resulted in 4261 highly variable and expressed genes. To map the high dimensional nature of the data matrix to a two-dimensional subspace we used t-SNE with an initial dimension of 30, a perplexity parameter of 30, 1000 maximum iterations and epoch parameter set to 100. We then used the dbscan algorithm (density-based spatial clustering) with eps = 5 and minPts = 15 to identify clusters. Thus, after steps-1 and 2, each cell is assigned to a unique potency state and co-expression cluster (cell-type).

Step-3 Identification of cell-states and construction of cell-density landscapes for each potency state: Specific potency-state single-cell cluster pairs may contain many cells and therefore represent clear candidates for defining cell-states. However, in principle, cells in the same state, whilst being in the same cluster, may not necessarily be that close in the tSNE embedding. For this reason, we also construct cell-density elevation maps for all single cells within each of the inferred potency states. In these surface maps, the elevation is directly proportional to cell-density. By comparing the resulting landscapes for each potency state, this may reveal novel cellular states characterized by high cell-density.

Step-4 Inference of bifurcations and lineage trajectories: From step-3, it is assumed that a cell-state of highest potency is identifiable. This provides a natural and unbiased way of assigning a root-state for subsequent application of a lineage-trajectory inference algorithm. We used Diffusion Maps^27^, as implemented in the destiny Bioconductor package^32^ with *k* = 30, otherwise default parameters were used. Pseudotime, specifically, DPT over the inferred trajectories was also computed using destiny.

### Estimation of cell-cycle and TPSC pluripotency scores

To identify single cells in either the G1-S or G2-M phases of the cell-cycle we followed the procedure described in ^5^. Briefly, genes whose expression is reflective of G1-S or G2-M phase were obtained from refs. ^64^^,^^65^. A given normalized scRNA-Seq data matrix for a given individual is then *z*-score normalized for all genes present in these signatures. Finally, a cycling score for each phase and each cell is obtained as the average *z*-scores over all genes present in each signature. When adjusting differential expression analyses for cell-cycle phase, we included the G1-S and G2-M scores as covariates in the linear models.

### Bulk expression datasets

In this study we used three mRNA expression datasets from bulk samples. One dataset consists of 38 FACS sorted bulk samples (Illumina expression beadarrays), as profiled by Shehata et al.^45^. Of the 38 samples, 10 were categorized as luminal non-clonogenic (L), i.e., terminally differentiated cells, with the rest (*n* = 28) making up a relatively differentiated (EpCAM+/CD49f + /ALDH-, *n* = 17) and undifferentiated (EpCAM+/CD49f + /ALDH+, *n* = 11) luminal progenitor (LP) populations. The two undifferentiated LP populations were further distinguished by expression or not of ERBB3. mRNA expression data was generated using Illumina Beadarrays and we used the normalized data, as described in ref. ^45^.

The second dataset is the METABRIC study, which profiled almost 2000 primary breast cancers using Illumina expression beadarrays^47^. We used the assignment of tumors to PAM50 intrinsic and integrative cluster subtypes as given by the METABRIC study. We used the normalized data, as provided by the METABRIC consortium.

A third Affymetrix mRNA expression dataset derives from Pece et al.^43^. This set consists of three separate pools of FACS sorted cell populations. Each pool contains a quiescent putative mammary stem cell population, as well as a population of derived progeny, consisting of transit-amplifying progenitor cells, thus a total of six bulk samples. We normalized the HGU133 plus2 data using the affy BioC package, specifically, the rma function. Only probes mapping to an Entrez gene ID were used, data was quantile normalized using limma, and probes mapping to the same gene were averaged, resulting in a normalized data matrix over 20,186 genes and six samples.

### Differential expression analysis

When performing differential expression analysis within the main single-cell clusters, differences in expression are smaller and therefore more susceptible to confounding by the technical dropout rate. Thus, when comparing gene expression of single-cell subgroups within a main single cell cluster, we always restrict to cells where the gene is expressed. That is, we remove all dropouts and do not impute data. When correlating to potency, we used a linear model between the normalized expression profile and the potency estimates, optionally adjusting for the two cell-cycle scores computed earlier. In the case of the Illumina beadarray datasets, we used the normalized data from the respective publications^45,47^ and called DE using the empirical Bayes limma framework^66^. We always use Bonferroni-adjusted thresholds to call statistical significance unless there are too few hits, in which case we relax the threshold using FDR < 0.05.

### Construction of the 144-gene bipotent signature and score

We performed differential expression analysis as described in previous section between the single-cells in the high potency (putative bipotent) single cell cluster to cells in the other two potency states using a linear model. A Bonferroni-adjusted *P* *<* 0.05 threshold was used to call significance. Because the great majority of differentially expressed genes were downregulated in the high potency state, with only 72 being upregulated, we defined a 144-gene signature consisting of the top 72 downregulated genes plus the 72 upregulated ones. The bipotency score in independent samples (e.g., METABRIC) was then obtained as the Pearson correlation of the signed 144-gene signature (i.e., using +1 for upregulated genes, and −1 for downregulated genes) with the expression profile of the independent sample.

### Doublet score analysis

We used two different simulation-based methods to derive doublet scores for each cell and to identify those more likely to be doublets. One approach used the simulation method of Dahlin et al.^40^ to obtain doublet scores for all single cells that passed QC and for each individual separately. Specifically, we used the doubletCells function (using approximate = TRUE option) from the *scran* R-package (version 1.10.1)^67^. In the second approach we used the Python package Scrublet^68^ (10.1101/357368). Within Scrublet, the scrub_doublets function, which is responsible for computing doublet scores and predicting doublets within a dataset, was run using default parameters.

### Statistics and reproducibility

All statistical analyses were performed with R version 3.6.0. *P*-values were estimated using Wilcoxon rank sum tests or linear regression, as indicated. Cox proportional hazards regression was used for survival analysis. Hazard Ratio, 95% confidence interval, and *P*-value as derived from the score-test is given for univariate analyses. In multivariate analysis, *P*-value derives from the Wald-test. We used the following open-source Bioconductor/R- packages: mclust_5.4.2, dbscan_1.1–3, tsne_0.1–3, igraph_1.2.4, monocle_2.99.3, scran_1.10.1, destiny_2.14.0.

### Reporting summary

Further information on research design is available in the Nature Research Reporting Summary linked to this article.

## Supplementary information

Reporting Summary

Description of additional supplementary items

Supplementary Data

Supplementary Information

## Data Availability

Data analyzed in this paper are already publicly available from the following GEO (www.ncbi.nlm.nih.gov/geo/) accession numbers: GSE113197, GSE35399, and GSE18931 or from the EGA (www.ebi.ac.uk/ega/) accession number EGAS00000000083. Source data for Figs. 2–7 are available as Supplementary Data. All other data supporting the findings of this study are available from the corresponding authors upon reasonable request.

## Code availability

LandSCENT^69^ is freely available as an R-package from github: https://github.com/ChenWeiyan/LandSCENT or from zenodo: https://zenodo.org/record/3257600 or from 10.5281/zenodo.3257600

## References

[1] Scialdone A (2016). Resolving early mesoderm diversification through single-cell expression profiling. Nature.

[2] Shepherd MS (2018). Single-cell approaches identify the molecular network driving malignant hematopoietic stem cell self-renewal. Blood.

[3] Laurenti E, Gottgens B (2018). From haematopoietic stem cells to complex differentiation landscapes. Nature.

[4] Trapnell C (2014). The dynamics and regulators of cell fate decisions are revealed by pseudotemporal ordering of single cells. Nat. Biotechnol..

[5] Tirosh I (2016). Dissecting the multicellular ecosystem of metastatic melanoma by single-cell RNA-seq. Science.

[6] Patel AP (2014). Single-cell RNA-seq highlights intratumoral heterogeneity in primary glioblastoma. Science.

[7] Haber AL (2017). A single-cell survey of the small intestinal epithelium. Nature.

[8] Tirosh I (2016). Single-cell RNA-seq supports a developmental hierarchy in human oligodendroglioma. Nature.

[9] Treutlein B (2014). Reconstructing lineage hierarchies of the distal lung epithelium using single-cell RNA-seq. Nature.

[10] Treutlein B (2016). Dissecting direct reprogramming from fibroblast to neuron using single-cell RNA-seq. Nature.

[11] Regev, A. et al. The Human Cell Atlas. Elife **6**, pii: e27041 (2017). 10.7554/eLife.27041 PMC5762154 29206104

[12] Hon CC, Shin JW, Carninci P, Stubbington MJT (2018). The Human Cell Atlas: technical approaches and challenges. Brief. Funct. Genom..

[13] Rozenblatt-Rosen O, Stubbington MJT, Regev A, Teichmann SA (2017). The Human Cell Atlas: from vision to reality. Nature.

[14] Grun D (2016). De novo prediction of stem cell identity using single-cell transcriptome data. Cell Stem Cell.

[15] Feinberg AP, Ohlsson R, Henikoff S (2006). The epigenetic progenitor origin of human cancer. Nat. Rev. Genet..

[16] Tomasetti C, Vogelstein B (2015). Cancer etiology. Variation in cancer risk among tissues can be explained by the number of stem cell divisions. Science.

[17] Tomasetti C, Li L, Vogelstein B (2017). Stem cell divisions, somatic mutations, cancer etiology, and cancer prevention. Science.

[18] Zhu L (2016). Multi-organ mapping of cancer risk. Cell.

[19] Trapnell C (2015). Defining cell types and states with single-cell genomics. Genome Res..

[20] Yuan GC (2017). Challenges and emerging directions in single-cell analysis. Genome Biol..

[21] Stingl J (2006). Purification and unique properties of mammary epithelial stem cells. Nature.

[22] Costa F, Grun D, Backofen R (2018). GraphDDP: a graph-embedding approach to detect differentiation pathways in single-cell-data using prior class knowledge. Nat. Commun..

[23] Grun D (2018). Revealing routes of cellular differentiation by single-cell RNA-seq. Curr. Opin. Syst. Biol..

[24] Stegle O, Teichmann SA, Marioni JC (2015). Computational and analytical challenges in single-cell transcriptomics. Nat. Rev. Genet..

[25] Chen J, Schlitzer A, Chakarov S, Ginhoux F, Poidinger M (2016). Mpath maps multi-branching single-cell trajectories revealing progenitor cell progression during development. Nat. Commun..

[26] Marco E (2014). Bifurcation analysis of single-cell gene expression data reveals epigenetic landscape. Proc. Natl Acad. Sci. USA.

[27] Haghverdi L, Buttner M, Wolf FA, Buettner F, Theis FJ (2016). Diffusion pseudotime robustly reconstructs lineage branching. Nat. Methods.

[28] Qiu X (2017). Reversed graph embedding resolves complex single-cell trajectories. Nat. Methods.

[29] Cao J (2019). The single-cell transcriptional landscape of mammalian organogenesis. Nature.

[30] Teschendorff AE, Enver T (2017). Single-cell entropy for accurate estimation of differentiation potency from a cell’s transcriptome. Nat. Commun..

[31] Banerji CR (2013). Cellular network entropy as the energy potential in Waddington’s differentiation landscape. Sci. Rep..

[32] Angerer P (2016). Destiny: diffusion maps for large-scale single-cell data in R. Bioinformatics.

[33] Weinreb C, Wolock S, Tusi BK, Socolovsky M, Klein AM (2018). Fundamental limits on dynamic inference from single-cell snapshots. Proc. Natl Acad. Sci. USA.

[34] Nguyen QH (2018). Profiling human breast epithelial cells using single cell RNA sequencing identifies cell diversity. Nat. Commun..

[35] Colacino JA (2018). Heterogeneity of human breast stem and progenitor cells as revealed by transcriptional profiling. Stem Cell Rep..

[36] Shi, J., Teschendorff, A. E., Chen, W., Chen, L. & Li, T. Quantifying Waddington’s epigenetic landscape: a comparison of single-cell potency measures. *Brief Bioinform.* (2018). 10.1093/bib/bby093. 10.1093/bib/bby093 30289442

[37] Guo, M., Bao, E. L., Wagner, M., Whitsett, J. A. & Xu, Y. SLICE: determining cell differentiation and lineage based on single cell entropy. *Nucleic Acids Res.* **45**, e54 (2016). 10.1093/nar/gkw1278 PMC5397210 27998929

[38] van der Maaten L (2008). Visualizing data using t-SNE. J. Mach. Learn. Res..

[39] Ester, M., Kriegel, H. P., Sander, J. & Xu, X. A density-based algorithm for discovering clusters in large spatial databases with noise. In *Proc. 2nd International Conference on Knowledge Discovery and Data Mining (KDD-96)* (Institute for Computer Science, University of Munich, Munich, 1996).

[40] Dahlin JS (2018). A single-cell hematopoietic landscape resolves 8 lineage trajectories and defects in Kit mutant mice. Blood.

[41] Stingl J, Raouf A, Emerman JT, Eaves CJ (2005). Epithelial progenitors in the normal human mammary gland. J. Mammary Gland Biol. Neoplasia.

[42] Subramanian A (2005). Gene set enrichment analysis: a knowledge-based approach for interpreting genome-wide expression profiles. Proc. Natl Acad. Sci. USA.

[43] Pece S (2010). Biological and molecular heterogeneity of breast cancers correlates with their cancer stem cell content. Cell.

[44] Athanasiadis EI (2017). Single-cell RNA-sequencing uncovers transcriptional states and fate decisions in haematopoiesis. Nat. Commun..

[45] Shehata M (2012). Phenotypic and functional characterization of the luminal cell hierarchy of the mammary gland. Breast Cancer Res..

[46] Castro MA (2016). Regulators of genetic risk of breast cancer identified by integrative network analysis. Nat. Genet..

[47] Curtis Christina, Shah Sohrab P., Chin Suet-Feung, Turashvili Gulisa, Rueda Oscar M., Dunning Mark J., Speed Doug, Lynch Andy G., Samarajiwa Shamith, Yuan Yinyin, Gräf Stefan, Ha Gavin, Haffari Gholamreza, Bashashati Ali, Russell Roslin, McKinney Steven, Langerød Anita, Green Andrew, Provenzano Elena, Wishart Gordon, Pinder Sarah, Watson Peter, Markowetz Florian, Murphy Leigh, Ellis Ian, Purushotham Arnie, Børresen-Dale Anne-Lise, Brenton James D., Tavaré Simon, Caldas Carlos, Aparicio Samuel (2012). The genomic and transcriptomic architecture of 2,000 breast tumours reveals novel subgroups. Nature.

[48] Velten L (2017). Human haematopoietic stem cell lineage commitment is a continuous process. Nat. Cell Biol..

[49] Bach K (2017). Differentiation dynamics of mammary epithelial cells revealed by single-cell RNA sequencing. Nat. Commun..

[50] Davis FM (2016). Single-cell lineage tracing in the mammary gland reveals stochastic clonal dispersion of stem/progenitor cell progeny. Nat. Commun..

[51] Zhang DZ (2017). Basic transcription factor 3 is required for proliferation and epithelial-mesenchymal transition via regulation of FOXM1 and JAK2/STAT3 signaling in gastric cancer. Oncol. Res..

[52] Davies AH (2014). YB-1 transforms human mammary epithelial cells through chromatin remodeling leading to the development of basal-like breast cancer. Stem Cells.

[53] Lim E (2009). Aberrant luminal progenitors as the candidate target population for basal tumor development in BRCA1 mutation carriers. Nat. Med..

[54] Campbell TM, Castro MAA, de Oliveira KG, Ponder BAJ, Meyer KB (2018). ERalpha binding by transcription factors NFIB and YBX1 enables FGFR2 signaling to modulate estrogen responsiveness in breast cancer. Cancer Res..

[55] Celià-Terrassa Toni (2018). Mammary Stem Cells and Breast Cancer Stem Cells: Molecular Connections and Clinical Implications. Biomedicines.

[56] Banerji CR, Severini S, Caldas C, Teschendorff AE (2015). Intra-tumour signalling entropy determines clinical outcome in breast and lung cancer. PLoS Comput. Biol..

[57] Al-Hajj M, Wicha MS, Benito-Hernandez A, Morrison SJ, Clarke MF (2003). Prospective identification of tumorigenic breast cancer cells. Proc. Natl Acad. Sci. USA.

[58] Velasco-Velazquez MA, Popov VM, Lisanti MP, Pestell RG (2011). The role of breast cancer stem cells in metastasis and therapeutic implications. Am. J. Pathol..

[59] Tomasetti C, Vogelstein B (2015). Cancer risk: role of environment-response. Science.

[60] Yang Z (2016). Correlation of an epigenetic mitotic clock with cancer risk. Genome Biol..

[61] Teschendorff AE, Sollich P, Kuehn R (2014). Signalling entropy: a novel network-theoretical framework for systems analysis and interpretation of functional omic data. Methods.

[62] Cerami EG (2011). Pathway Commons, a web resource for biological pathway data. Nucleic Acids Res..

[63] Yeung KY, Fraley C, Murua A, Raftery AE, Ruzzo WL (2001). Model-based clustering and data transformations for gene expression data. Bioinformatics.

[64] Whitfield ML (2002). Identification of genes periodically expressed in the human cell cycle and their expression in tumors. Mol. Biol. Cell.

[65] Macosko EZ (2015). Highly parallel genome-wide expression profiling of individual cells using nanoliter droplets. Cell.

[66] Smyth Gordon K (2004). Linear Models and Empirical Bayes Methods for Assessing Differential Expression in Microarray Experiments. Statistical Applications in Genetics and Molecular Biology.

[67] Lun AT, McCarthy DJ, Marioni JC (2016). A step-by-step workflow for low-level analysis of single-cell RNA-seq data with bioconductor. F1000Res.

[68] Wolock, S. L., Lopez, R. & Klein, A. M. Scrublet: computational identification of cell doublets in single-cell transcriptomic data. *bioRxiv* (2018). 10.1016/j.cels.2018.11.005 PMC6625319 30954476

[69] Chen, W. & Teschendorff, A. E. LandSCENT package 10.5281/zenodo.3257600. (2019).

