# Supplementary material for: Single-cell landscape in mammary epithelium reveals bipotent-like cells associated with breast cancer risk and outcome
Source: Commun Biol. 2019 Aug 9;2:306. doi: 10.1038/s42003-019-0554-8 (PMC6689007; doi:10.1038/s42003-019-0554-8)
Supplement: Supplementary file 2 — Description of additional supplementary items [file 42003_2019_554_MOESM2_ESM.docx]

Description of additional supplementary items

Title: Source Data

Description: The underlying data for figures 2-7.
