# Supplementary material for: Single-cell landscape in mammary epithelium reveals bipotent-like cells associated with breast cancer risk and outcome
Source: Commun Biol. 2019 Aug 9;2:306. doi: 10.1038/s42003-019-0554-8 (PMC6689007; doi:10.1038/s42003-019-0554-8)
Supplement: Supplementary file 4 — Supplementary Information [file 42003_2019_554_MOESM4_ESM.pdf]

SUPPLEMENTARY FIGURES

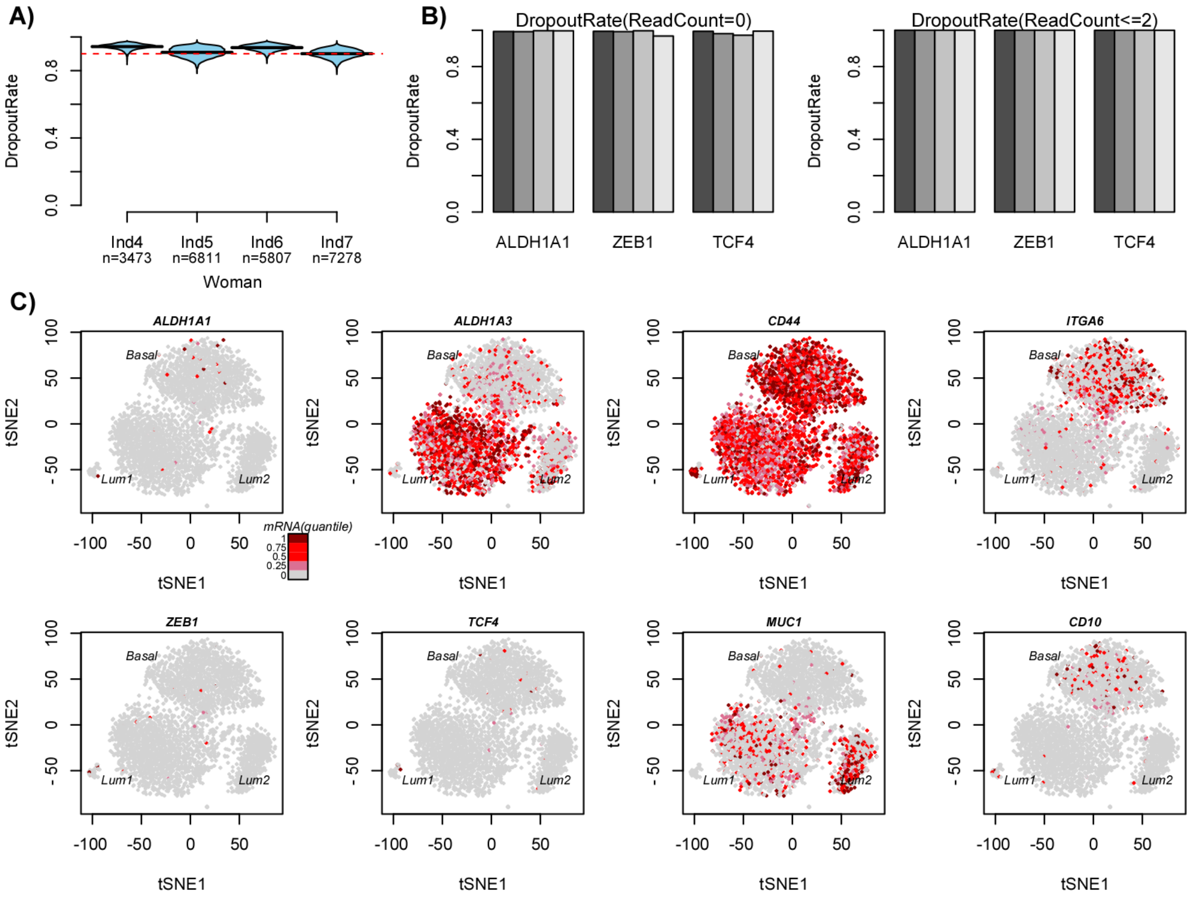

Supplementary Figure 1: High Dropout Rate in 10X scRNA-Seq data from mammary epithelium. A) Violin plots of the overall dropout rate (y-axis) for each of the 4 women (x-axis), with the number of cells (after QC) indicated below the women-ID. Red dashed line indicates 90% dropout rate. **B)** The dropout rate for 3 proposed stemness markers (*ALDH1A1*, *ZEB1* and *TCF4*) across single cells and for each of the 4 women (different shades of gray) and for two different definitions of dropout: left panel calls dropouts where the number of reads is zero, whereas the right panel considers dropouts to be read counts less or equal than 2. The precise values for the left panel are (from left to right): 0.993, 0.992, 0.997, 0.997, 0.994, 0.992, 0.997, 0.968, 0.994, 0.981, 0.972, 0.995) and for the right panel (from left to right): 1, 1, 1, 1, 0.9997, 1, 1, 1, 1, 0.9997, 0.9997, 1, 0.9999. **C)** t-SNE scatterplots for the scRNA-Seq from Ind-4, with single cells color-labeled according to the expression of specific stemness/progenitor markers, as shown.

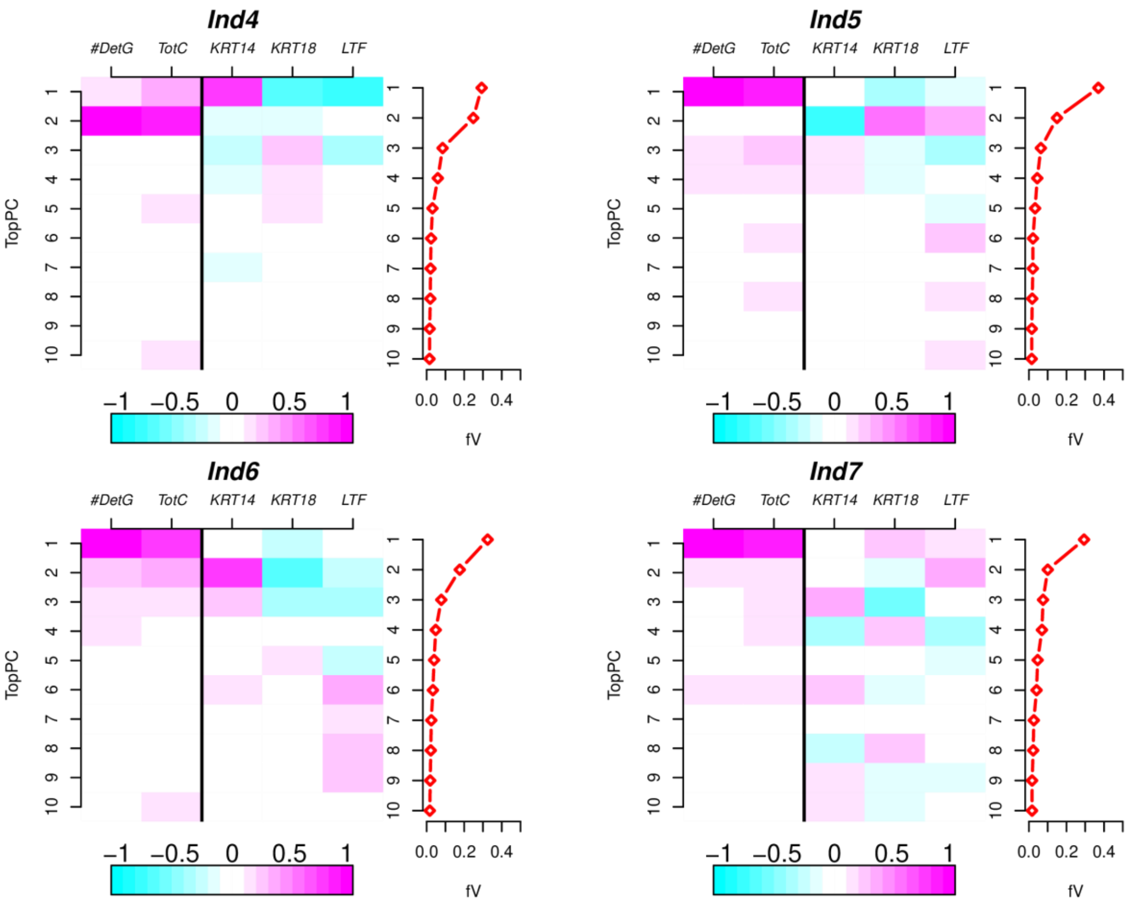

Supplementary Figure 2: Quality Control PCA correlation analysis. For each of the 4 women, we display a Pearson Correlation Coefficient heatmap between the top 10 principal components (TopPC) and 5 variables, including the number of detected genes per cell (*#DetG*), the total read count (<u>*TotC*</u>) and the normalized expression levels of *KRT14* (a basal marker), *KRT18* (a luminal marker) and *LTF* (lactotransferin- a marker of immature luminal cells). The right panel displays the fraction of explained variance (fV, x-axis) of each top PC. For each woman, additional PCs that were deemed to carry significant variance according to our RMT estimation procedure [1] have been suppressed, but these don't show significant correlations.

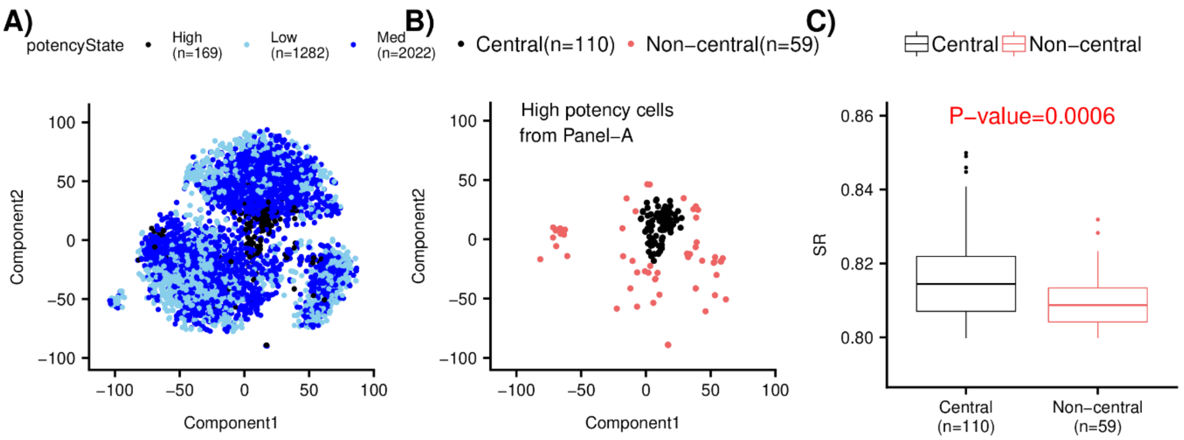

Supplementary Figure 3: Highest entropy/potency cells occupy topologically central position. A) t-SNE neighborhood plot for the 3473 single cells, with cells colored according to their inferred potency class. **B)** As A), but now only displaying the cells in the highest potency class, with colors now labeling de-novo clusters inferred using DBSCAN. **C)** Comparison of entropy SR values (cell potency values) between the high-potency cells in the central cluster vs the high-potency cells that mapped elsewhere (non-central). P-value is from a one-tailed Wilcoxon rank sum test.

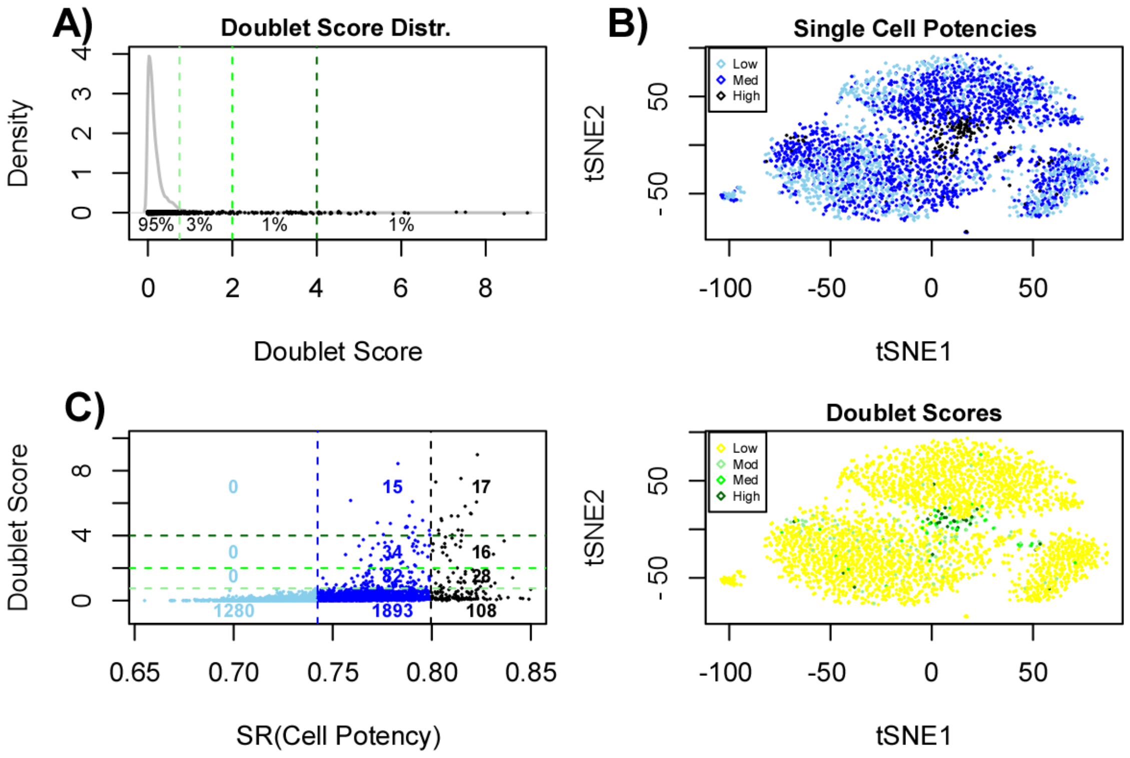

Supplementary Figure 4: Doublet analysis using scran (Dahlin et al [2]). A) Density distribution of doublet scores of the 3473 single cells from individual-4, with the proportions of cells falling within different score-bin categories. Doublet scores were obtained using the *scran* R-package. **B)** tSNE plot for all 3473 single cells, displaying their inferred potency states (upper panel) and their binned doublet scores (lower panel), as shown. **C)** Scatterplot of the doublet scores (y-axis) against signaling entropy rate (SR, x-axis) for all 3473 single cells. Dashed lines indicate the boundaries defining potency states and binned doublet scores, and the numbers indicate the number of cells falling within each rectangular bin.

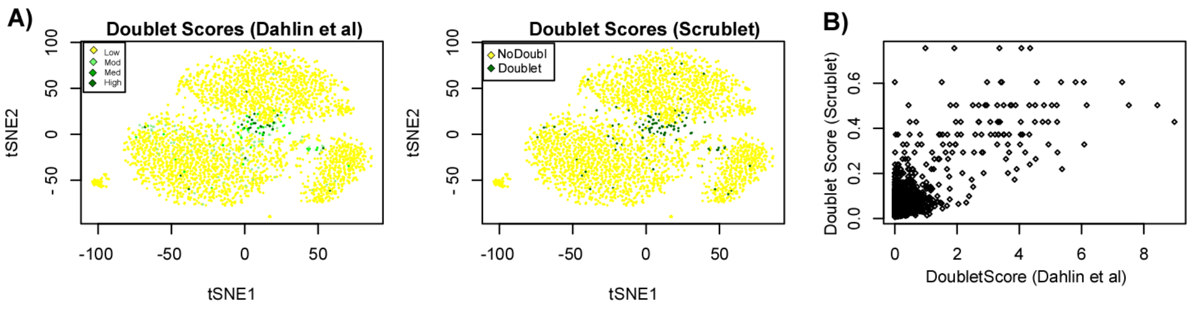

Supplementary Figure 5: Doublet analysis using Scrublet [3]. A) tSNE plots for all 3473 single cells from individual-4, displaying their doublet scores according to scran (left panel) and scrublet (right panel). **B)** Scatterplot of the doublet scores according to scran (x-axis) against those of scrublet (y-axis) for all 3473 single cells.

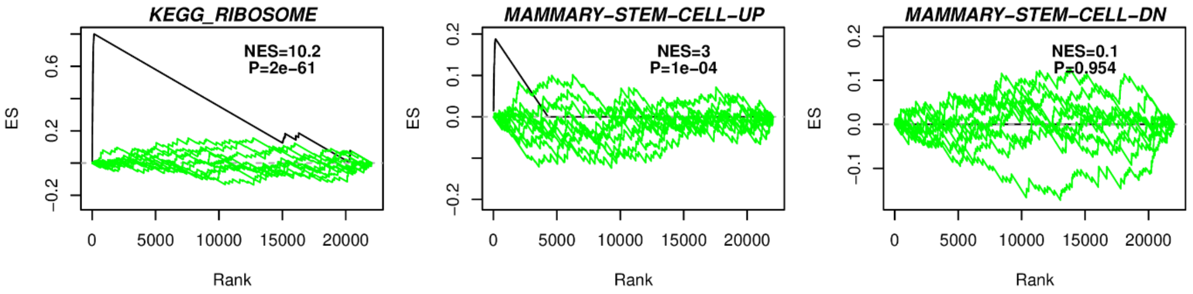

Supplementary Figure 6: Rank-based GSEA reveals correlation of mammary stem-cell and ribosomal protein modules with increased potency in mammary epithelium. Plots of the Enrichment Score (ES, y-axis) from rank-based GSEA against rank index position (x-axis) for genes ranked according to their positive correlation with potency as assessed using the scRNA-Seq data (black line), and for three different biological terms from the MSigDB dataset: Ribosomal genes from the KEGG database, genes upregulated in mammary stem cells (Pece et al) and genes downregulated in mammary stem cells (Pece et al [4]). Green curves describe dependence of the ES score on rank position after Monte-Carlo randomization of the gene-ranking, for 10 different Monte-Carlo runs. The Normalized Enrichment Score (NES) defined by the ratio of the observed maximum ES score to the mean of the maximum over 1000 Monte-Carlo runs is given, as well as the associated P-value derived by approximating the max ES scores over the 1000 Monte-Carlo runs as a Gaussian.

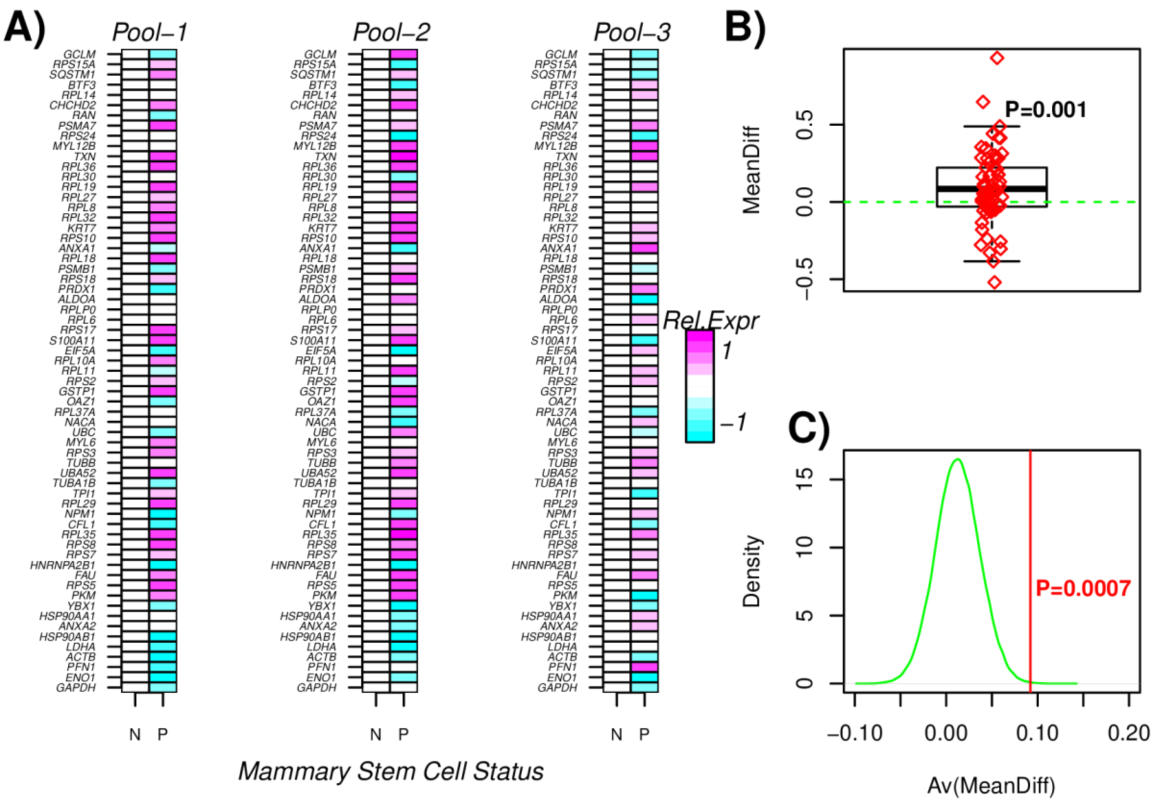

Supplementary Figure 7: Stem-like single-cell expression signature is increased in mammary stem cell pools. A) Normalized relative expression heatmaps for 63 represented genes from the 72 genes upregulated in the putative stem-like single-cells, in 3 separate pools of FACS sorted quiescent mammary stem-cells (P) and their derived proliferative non-stem like progeny (N). **B)** Average expression difference between the P and N cells, averaged over the 3 separate pools. P-value is from a one-tailed Wilcoxon rank sum test. **C)** Monte-Carlo randomization analysis, where in each of 100,000 random selections of 63 genes, the average difference over the 3 pools is computed (green curve) and compared to the observed average difference (red line). Monte-Carlo P-value is given.

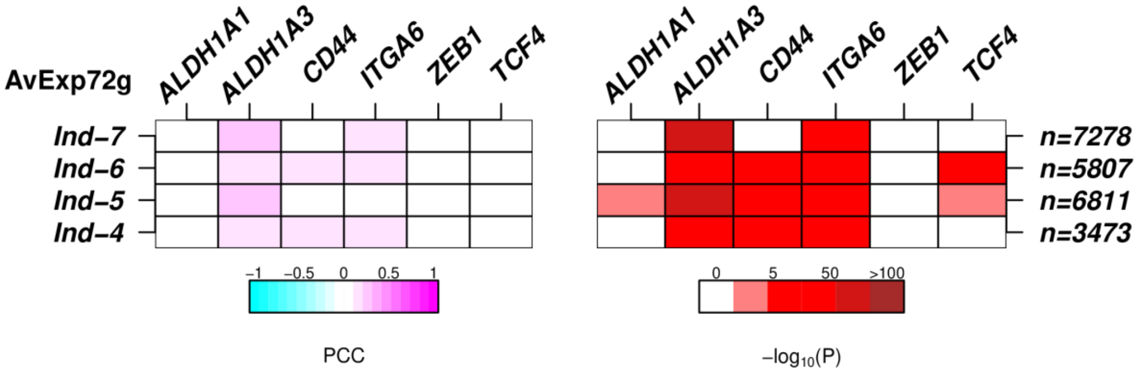

Supplementary Figure 8: Correlation of single-cell stem-like signature with stemness marker expression in 10X scRNA-Seq dataset. Heatmap of Pearson correlation coefficients (PCC) between the average expression of all 72 genes (AvExp72g) making up the single-cell stem-like signature with the expression of 6 stemness markers (*ALDH1A1*, *ALDH1A3*, *CD44*, *ITGA6*, *ZEB1* and *TCF4*), as indicated. The correlations were computed in the 10X scRNA-Seq dataset, separately for the four different women as indicated. Right panel heatmap displays the corresponding $-\log_{10}$ [P-values], and the number of cells over which the correlations and P-values were estimated are given on the right.

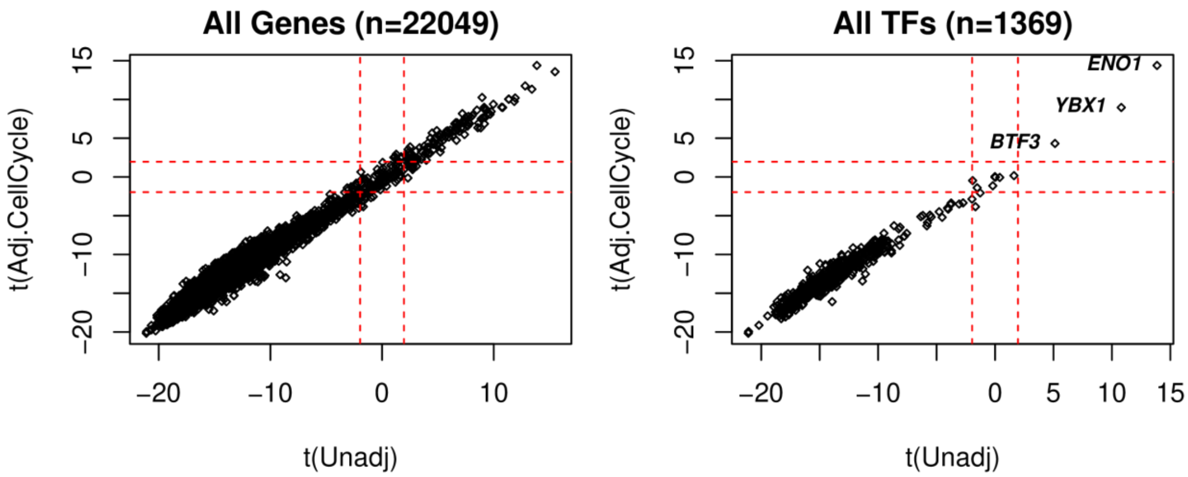

Supplementary Figure 9: Differential expression potency analysis adjusted for cell-cycle phase. Scatterplots of t-statistics of association with signaling entropy rate and gene expression unadjusted for cell-cycle phase (x-axis) vs adjusted for cell-cycle phase (y-axis) for all genes (left panel) and for all transcription factors (TFs) (right panel).

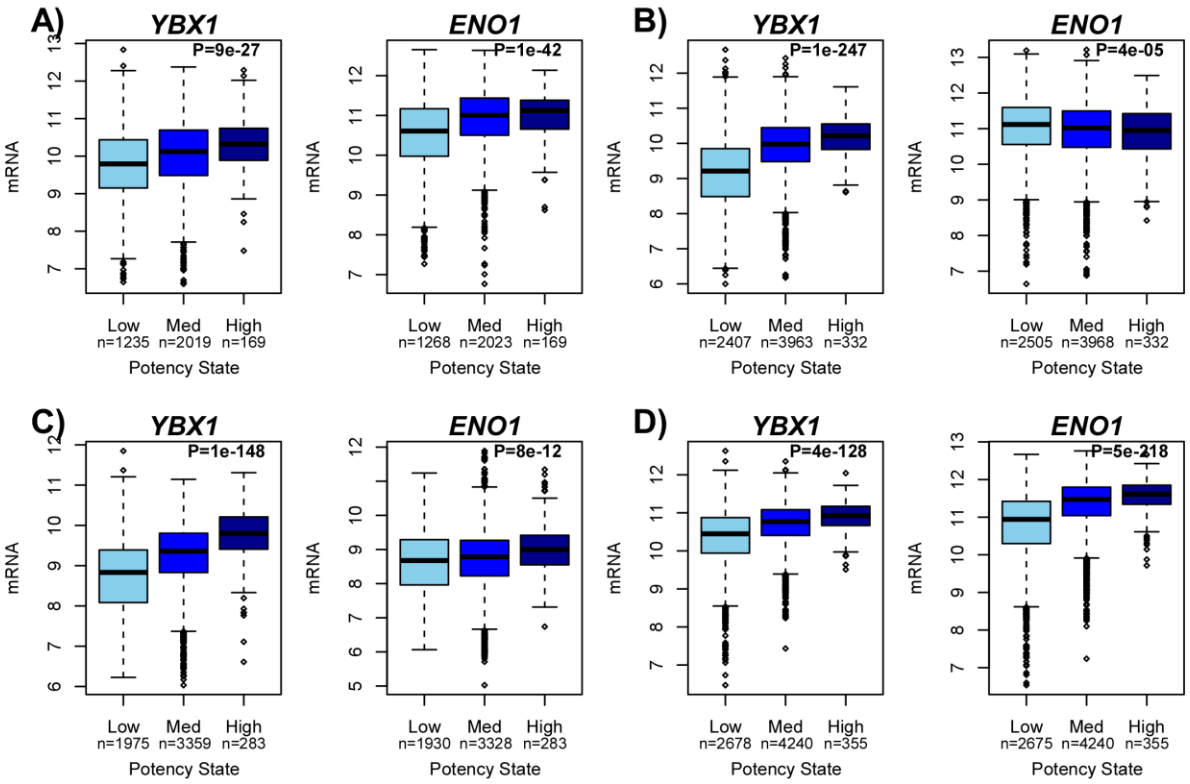

Supplementary Figure 10: *YBX1* and *ENO1* expression correlate with potency. A) Boxplots of normalized log-expression (y-axis) for *YBX1* and *ENO1* against inferred potency state (x-axis) for all single cells where these genes were expressed. Numbers of single-cells assigned to each potency state is given. P-value is from a two-tailed linear regression. All single-cell cells derive from one individual (Ind-4). **B, C, D)** As A), but for other 3 women (Ind-5,6,7, respectively).

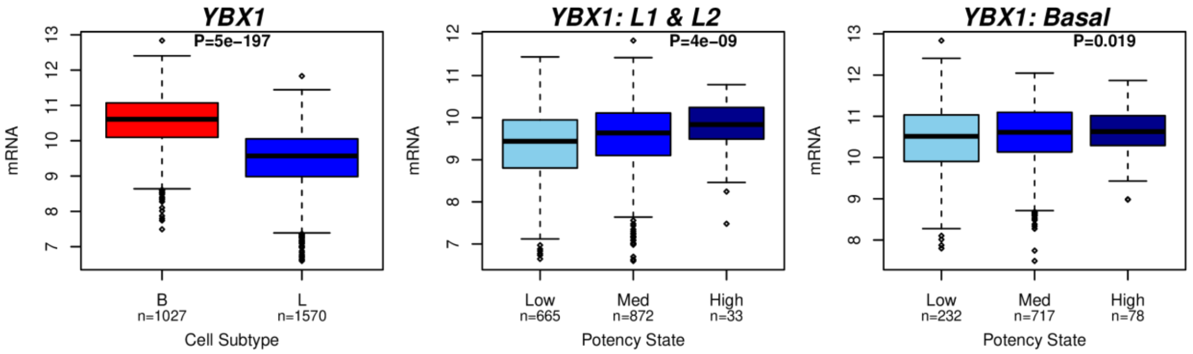

Supplementary Figure 11: *YBX1* expression across the basal luminal divide and potency states. Left panel: Boxplot of expression of *YBX1* between the single-cells from the basal cluster (B) and those of the two luminal (L1 & L2) clusters. P-value is from a t-test and number of cells in each category is given below boxplot. **Middle & right panels:** Boxplot of *YBX1* expression against potency state restricting to luminal and basal cells, respectively. P-value is from a linear regression t-test and number of cells in each category is given below boxplot. In all panels, only cells expressing *YBX1* were included.

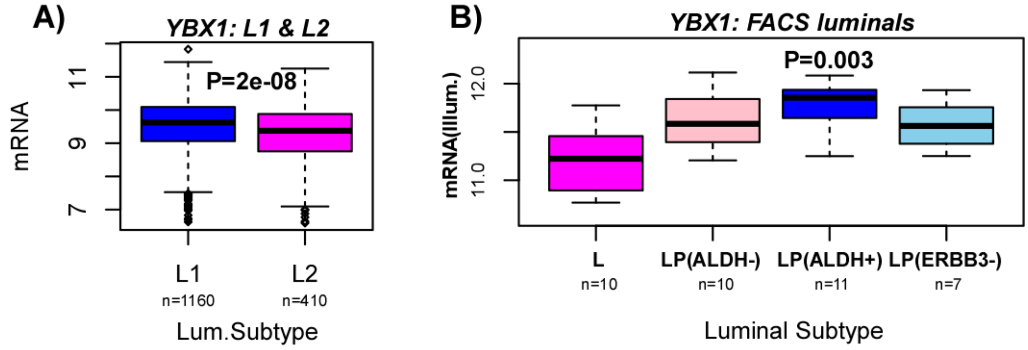

Supplementary Figure 12: *YBX1* expression correlates with luminal progenitor status. A) Boxplot of expression of *YBX1* between the single-cells from the two luminal (L1 & L2) clusters, with L2 denoting the mature luminal phenotype. P-value is from a t-test and number of cells in each category is given. **B)** Boxplot of mRNA expression (Illumina Beadarray) of *YBX1* for luminal cells (L) and 3 putative luminal progenitor (LP_ subpopulations, as described in Shehata et al. P-value is from a one-tailed Wilcoxon rank sum test comparing the ALDH+ subpopulation to all the rest.

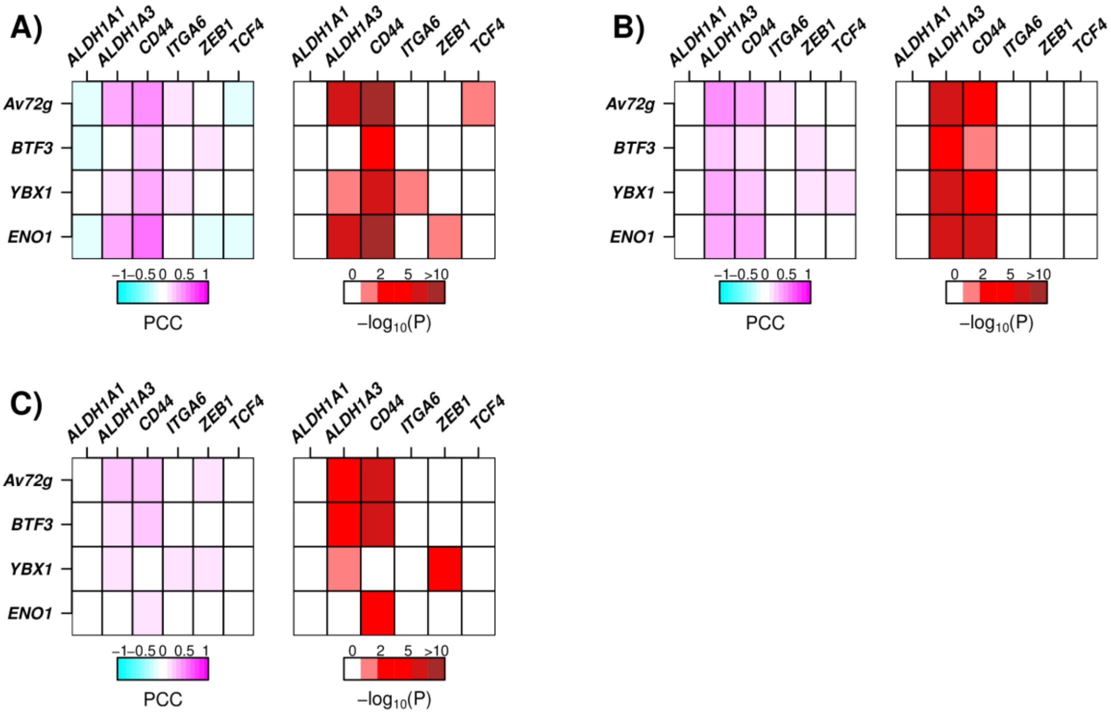

Supplementary Figure 13: Correlation of bipotent single-cell signature genes with stemness markers in Fluidigm C1 dataset. A) Heatmap of Pearson correlation coefficients (PCC) between the expression of *ENO1*, *YBX1*, *BTF3* and the average of all 72 genes (Av72g) in the bipotent stem-like signature with the expression of 6 stemness markers (*ALDH1A1*, *ALDH1A3*, *CD44*, *ITGA6*, *ZEB1* and *TCF4*) as indicated. The correlations are computed in the Fluidigm C1 scRNA-Seq dataset from one woman (Ind-1, n=198 cells) from Nguyen et al [5]. Right panel heatmap displays the corresponding $-\log_{10}$ [P-values]. **B-C)** As A, but for the other 2 women: Ind-2 (n=195), Ind-3 (n=322).

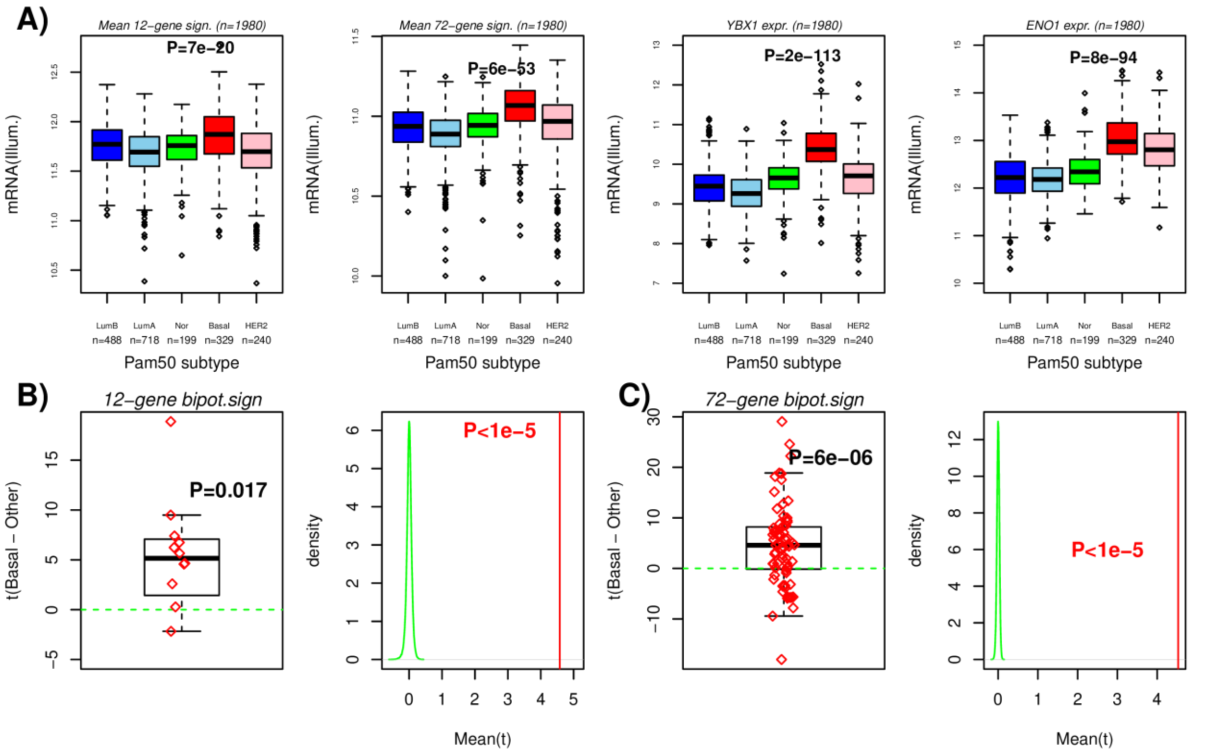

Supplementary Figure 14: Association of bipotent single-cell expression signature with basal breast cancer in METABRIC [6]. A) Boxplots of the mean expression of the 12-gene and 72-gene bipotent signatures, as well as *YBX1* and *ENO1* expression across the PAM50 subtypes in the METABRIC cohort. P-values are from a Wilcoxon test between the basal group and all others. Number of samples in each subtype are indicated. Darkblue=lum-B, skyblue=lum-A, green=normal-like, red=basal, pink=HER2+. **B)** Boxplot of t-statistics of differential expression for the 12-gene bipotent signature. P-value is from a one-tailed Wilcox test. Monte-Carlo randomization (100,000 runs) comparing the mean t-statistic for 12 randomly selected genes (green curve) against the observed average (red vertical line). **C)** As B) but for the 72 upregulated genes in the bipotent signature.

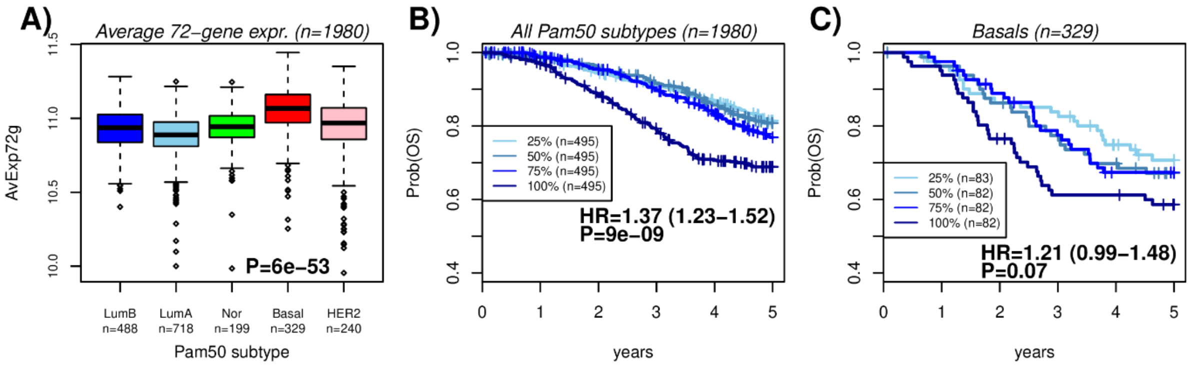

Supplementary Figure 15: Association of bipotent single-cell expression signature with clinical outcome in METABRIC. A) Boxplot of the average expression over the 72 upregulated genes in the stem-like single cell signature vs breast cancer intrinsic subtype in the METABRIC cohort. P-value is from a Wilcox test comparing basals to all others. Number of samples in each subtype are given. **B)** Kaplan Meier overall survival curves for all 1980 METABRIC samples, stratified by quartiles of the average 72 gene expression, and with survival data censored at 5 years after diagnosis. Hazard Ratio (HR), 95% CI and P-value are from a Cox proportional hazards regression. **C)** As B), but now focusing only on the 328 basal samples.

SUPPLEMENTARY TABLES

	Entrez ID	t	P	n
GAPDH	2597	15.49	2.00E-52	3452
ENO1	2023	13.86	2.00E-42	3460
PFN1	5216	13.41	5.00E-40	3430
ACTB	60	12.81	1.00E-36	3425
LDHA	3939	11.92	4.00E-32	3329
HSP90AB1	3326	11.84	1.00E-31	3412
ANXA2	302	11.35	2.00E-29	3402
HSP90AA1	3320	11.33	3.00E-29	3334
YBX1	4904	10.8	9.00E-27	3423
PKM	5315	10.71	2.00E-26	3361
RPS5	6193	9.96	4.00E-23	3465
FAU	2197	9.77	3.00E-22	3460
HNRNPA2B1	3181	9.67	7.00E-22	3369
RPS7	6201	9.48	4.00E-21	3469
RPS8	6202	9.35	2.00E-20	3470
RPL35	11224	9.22	5.00E-20	3469
RPSA	3921	9.17	8.00E-20	3397
CFL1	1072	9.16	9.00E-20	3323
RPL7A	6130	9.14	1.00E-19	3457
NPM1	4869	9.03	3.00E-19	3431
RPL29	6159	8.95	6.00E-19	3467
TPI1	7167	8.88	1.00E-18	3266
TUBA1B	10376	8.83	2.00E-18	3219
UBA52	7311	8.65	8.00E-18	3410
TUBB	203068	8.58	1.00E-17	3382
RPS3	6188	8.5	3.00E-17	3473
RPL5	6125	8.08	9.00E-16	3456
MYL6	4637	7.95	2.00E-15	3430
UBC	7316	7.84	6.00E-15	3426
NACA	4666	7.74	1.00E-14	3464
RPL37A	6168	7.73	1.00E-14	3470
OAZ1	4946	7.67	2.00E-14	3440
GSTP1	2950	7.66	2.00E-14	3408
RPS2	6187	7.45	1.00E-13	3473
RPL11	6135	7.41	2.00E-13	3471
RPL10A	4736	7.41	2.00E-13	3458
EIF5A	1984	7.38	2.00E-13	3190
ACTG1	71	7.36	2.00E-13	3267
RPL4	6124	7.34	3.00E-13	3415

S100A11	6282	7.3	4.00E-13	3462
RPS17	6218	7.24	5.00E-13	3468
RPL6	6128	7.19	8.00E-13	3469
RPLP0	6175	7.06	2.00E-12	3471
ALDOA	226	6.93	5.00E-12	3289
PRDX1	5052	6.88	7.00E-12	3426
RPL28	6158	6.76	2.00E-11	3458
RPS18	6222	6.71	2.00E-11	3473
PSMB1	5689	6.66	3.00E-11	3277
RPL18	6141	6.61	4.00E-11	3465
ANXA1	301	6.6	5.00E-11	3084
RPS10	6204	6.56	6.00E-11	3379
KRT7	3855	6.51	9.00E-11	3228
RPL32	6161	6.36	2.00E-10	3473
RPL23	9349	6.3	3.00E-10	3365
RPL8	6132	6.1	1.00E-09	3472
RPL27	6155	5.98	2.00E-09	3395
RPL19	6143	5.96	3.00E-09	3472
RPL30	6156	5.86	5.00E-09	3443
RPS3A	6189	5.86	5.00E-09	3468
RPL36	25873	5.75	1.00E-08	3461
TXN	7295	5.67	2.00E-08	3448
MYL12B	103910	5.51	4.00E-08	3381
RPS24	6229	5.4	7.00E-08	3456
PSMA7	5688	5.31	1.00E-07	3243
RAN	5901	5.21	2.00E-07	3097
CHCHD2	51142	5.17	2.00E-07	3407
RPL14	9045	5.14	3.00E-07	3466
BTF3	689	5.13	3.00E-07	3405
SQSTM1	8878	4.97	7.00E-07	3376
RPS15A	6210	4.9	1.00E-06	3459
GCLM	2730	4.89	1.00E-06	3320
RPL18A	6142	4.74	2.00E-06	3471
SURF1	6834	-20.76	2.00E-83	1383
ASH1L	55870	-21.08	1.00E-80	910
RSRP1	57035	-20.64	3.00E-80	1103
CD46	4179	-20.14	1.00E-79	1435
PTPMT1	114971	-20.66	2.00E-77	868
DUT	1854	-19.66	4.00E-77	1585
CDC26	246184	-19.71	2.00E-76	1403
CHURC1	91612	-19.69	2.00E-76	1400
HP1BP3	50809	-19.69	4.00E-76	1355
RNASEK	440400	-20.28	9.00E-76	917

UNC50	25972	-19.71	1.00E-75	1278
GABARAP	11337	-19.77	1.00E-75	1207
SRPRA	6734	-19.8	2.00E-75	1166
PET100	1E+08	-19.35	4.00E-75	1605
ARL6IP1	23204	-19.65	7.00E-75	1221
ITGB8	3696	-19.77	9.00E-75	1113
NEU1	4758	-19.4	1.00E-74	1438
SERINC3	10955	-19.5	1.00E-74	1323
EIF1B	10289	-19.35	2.00E-74	1467
C6orf48	50854	-19.34	6.00E-74	1393
DPM3	54344	-19.53	3.00E-73	1111
SLC50A1	55974	-19.5	9.00E-73	1080
NUCB1	4924	-19.22	9.00E-73	1320
TMEM256	254863	-19.57	1.00E-72	1027
BLOC1S4	55330	-19.82	1.00E-72	891
APOPT1	84334	-19.76	1.00E-72	921
PHF14	9678	-19.41	4.00E-72	1065
IFNGR2	3460	-19.03	8.00E-72	1375
RERE	473	-21.11	1.00E-71	517
RSF1	51773	-19.22	2.00E-71	1141
ARFGAP3	26286	-18.98	2.00E-71	1380
TMED5	50999	-19.17	2.00E-71	1183
TMEM106C	79022	-18.9	2.00E-71	1452
CHMP3	51652	-19.45	2.00E-71	963
DSP	1832	-19.21	3.00E-71	1114
BCL6	604	-20.2	3.00E-71	673
LSM14A	26065	-18.92	4.00E-71	1377
MRPL42	28977	-20.15	4.00E-71	678
GABPB1	2553	-19.14	5.00E-71	1150
UBAC2	337867	-19.04	5.00E-71	1238
ANAPC5	51433	-19.6	5.00E-71	866
CRK	1398	-19.79	8.00E-71	775
DPP7	29952	-18.94	1.00E-70	1276
PMPCB	9512	-18.8	1.00E-70	1420
HBP1	26959	-18.82	2.00E-70	1379
LSM8	51691	-19.21	2.00E-70	1022
CLIP1	6249	-19.2	2.00E-70	1028
ARL5A	26225	-19.04	2.00E-70	1140
TRMT11	60487	-19.51	3.00E-70	845
EAPP	55837	-18.88	3.00E-70	1261
MRPL54	116541	-19.51	3.00E-70	842
COQ10B	80219	-18.91	4.00E-70	1225
OPTN	10133	-18.79	4.00E-70	1351

SMDT1	91689	-18.5	4.00E-70	1805
METTL23	124512	-19.19	5.00E-70	995
MBTPS1	8720	-20.36	5.00E-70	586
NSA2	10412	-18.99	6.00E-70	1122
ERLEC1	27248	-19.02	6.00E-70	1100
NDUFA7	4701	-19.39	7.00E-70	871
B4GALT3	8703	-19.27	7.00E-70	932
TMEM132A	54972	-19.05	8.00E-70	1069
METTL9	51108	-18.64	8.00E-70	1487
ATP2A2	488	-19.25	9.00E-70	935
TSPAN31	6302	-19.61	1.00E-69	768
PSENEN	55851	-18.62	1.00E-69	1470
ZSWIM7	125150	-19.65	2.00E-69	745
GTF2I	2969	-18.92	2.00E-69	1127
OSBPL9	114883	-20.14	2.00E-69	609
CCDC186	55088	-19.19	2.00E-69	939
TEX264	51368	-19.21	2.00E-69	917
TARDBP	23435	-19.03	3.00E-69	1016
MCUR1	63933	-18.74	3.00E-69	1257

Supplementary Table 1. The top 72 upregulated and 72 downregulated genes in the high-potency single-cell cluster. Table lists the gene symbol, Entrez gene ID, the t-statistic (t) of differential expression, the corresponding P-value (P) and the number of cells (n) expressing the given gene and used in deriving the t-statistic and P-value. Upregulated genes are indicated in red, downregulated genes in black. All genes pass a Bonferroni adjusted P<0.05 threshold.

	coef	HR(=exp(coef))	se(coef)	z	Pr(>|z|)
BipotentScore	0.23	1.26	0.07	3.42	6.00E-04
ER-status	-0.73	0.48	0.14	-5.22	2.00E-07
Age	0.3	1.35	0.06	4.81	2.00E-06
Grade	0.41	1.51	0.12	3.59	3.00E-04
Stage	0.2	1.23	0.06	3.49	5.00E-04
Size	0.22	1.24	0.04	5.74	1.00E-08

Supplementary Table 2: Multivariate Cox-analysis for 144-gene bipotent signature in all METABRIC sample (n=1980). Table lists the covariate, the estimated Cox-regression coefficient, the Hazard Ratio (HR), the standard error in the regression coefficient, the Wald z-statistic and the corresponding P-value. Covariates include the bipotency score (from the 144-gene bipotent signature), estrogen receptor (ER) status, age, grade, stage and tumor size.

	coef	exp(coef)	se(coef)	z	Pr(>|z|)
BipotentScore	0.26	1.3	0.12	2.16	0.031
Age	0.03	1.03	0.12	0.23	0.817
Stage	0.11	1.12	0.12	0.94	0.349
Size	0.33	1.4	0.1	3.26	0.001

Supplementary Table 3: Multivariate Cox-analysis for 144-gene bipotent signature in METABRIC basal samples (n=329). Table lists the covariate, the estimated Cox-regression coefficient, the Hazard Ratio (HR), the standard error in the regression coefficient, the Wald z-statistic and the corresponding P-value. Covariates include the stemness/bipotency score (from the 144-gene bipotent signature), estrogen receptor (ER) status, age, grade, stage and tumor size.

SUPPLEMENTARY NOTES

Validation of potency assignments

Although our single-cell potency (SR) measure has been extensively validated on both single-cells and bulk samples [7,8], we sought additional validation of the specific potency assignments in the current 10X scRNA-Seq dataset. It is well known that genes like *GATA3*, *FOXA1* and *ESR1* are associated with a more differentiated luminal phenotype and therefore the expectation would be that their expression levels should be higher in the luminal cells of lowest potency. Because we are here interested in detecting relatively smaller expression changes, we limited the confounding effect of technical dropouts by assessing differential expression (DE) using only cells that express the given gene. This DE calling strategy itself requires a validation before we can implement it to test our potency assignments.

In order to validate the DE calling strategy, we considered a scenario where expression differences are larger, e.g. as between basal and luminal cells. To this end, we used bulk mRNA expression data from FACS sorted differentiated luminal and basal cells [9] to define a gold-standard list of 5,773 differentially expressed genes (DEGs) between basal and luminal cells. This was done as described in Methods section. The expectation is that these genes should exhibit corresponding differential expression changes between the basal and luminal single cell clusters. Thus, for each of the gold-standard genes, and using only cells expressing the corresponding gene, we derived a t-statistic of differential expression between the single cell basal and luminal clusters, which revealed that for the great majority of gold-standard DEGs, these exhibited the expected pattern of differential expression (OR=8.31, Fisher=test P=2e-26), as depicted in the figure below:

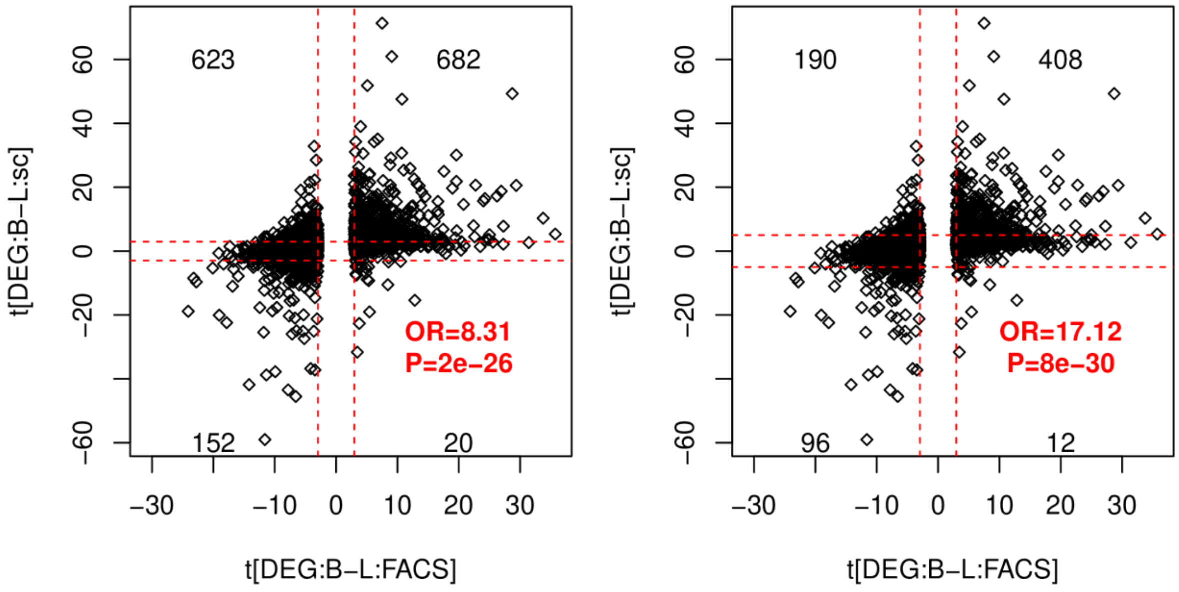

Validation of DEG calling method using independent FACS bulk data. *Each panel depicts a scatterplot of the t-statistics of differential expression between FACS sorted basal and luminal cell populations (Illumina beadarray data) (x-axis) against the corresponding t-statistics of differential expression between the single-cell basal and luminal (L1&L2) clusters (y-axis), where dropouts have been removed (i.e. only single cells with expression values have been used). The red dashed vertical lines indicate the FDR=0.05 threshold, whereas the horizontal lines indicate t-tstatistic thresholds of +/- 2.96 (left panel) and +/- 5 (right panel), corresponding to P-values of 0.3 and 0.004, respectively. Odds Ratios and one-tailed Fisher-exact test P-values are given.*

Having validated our method for calling DE, we next confirmed the lower expression of differentiation markers like *GATA3* in the more potent luminal cells (see figure below). We also validated the potency assignments within the basal compartment. For instance, we observed that expression of *KRT5* and *EGFR*, two well-known basal differentiation markers, decreased in the basal cells of higher potency:

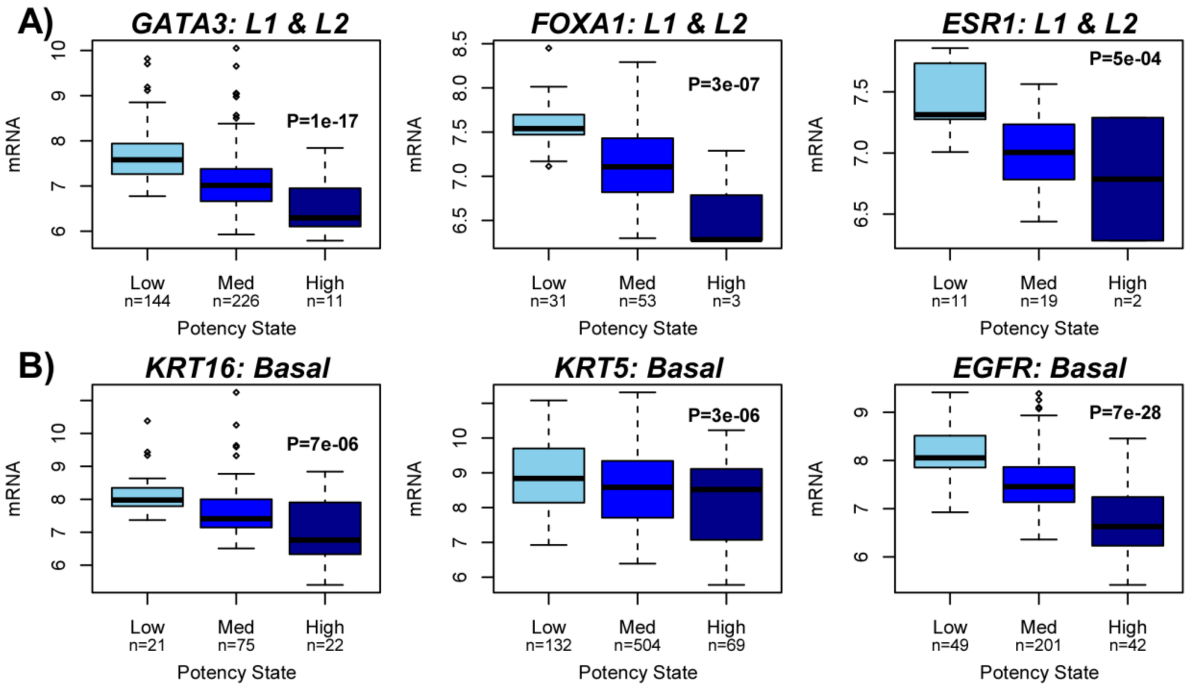

Validation of potency assignments. A) *Boxplots of normalized log-expression (y-axis) for known markers of luminal differentiated cells (GATA3, FOXA1) and hormone receptor (ESR1) against inferred potency state (x-axis) for all single cells assigned to the two main luminal clusters (L1 & L2) and further restricting to cells where these genes are expressed. Numbers of single-cells assigned to each potency state is given. P-value is from a (two-tailed) linear regression.* ***B)*** *As A), but for known basal differentiation markers (KRT16, KRT5, EGFR) and*

restricting to cells that were assigned to the basal cluster.

Competing analysis methods do not identify a bipotent stem-like state

We asked if LandSCENT is critical to the identification of the bipotent stem-like state. To this end, we re-analyzed the 10X dataset using a state-of-the-art algorithm called Monocle-2 [10]. Details of parameters used are given in Methods section. Although this algorithm is designed for timecourse data, it has also been widely used to estimate pseudotime and infer lineage-trajectories in non-timecourse data such as the 10X dataset considered here. Using Monocle-2 we largely reproduced the clustering, with effectively 3 main clusters (basal, immature luminal and luminal) and additional clusters mapping to the joint peripheral areas (see **panel-A** in figure below). With Monocle-2 we also inferred a lineage trajectory through the main clusters (**panel-A**). Estimation of pseudotime or "differentiation potency" within Monocle-2 however, requires specification of a "root-state" to define the origin of pseudotime, and Monocle-2 can only infer this root state via prior biological knowledge such as putative stemness markers. Considering the previous panel of 6 stemness markers (*ALDH1A1*, *ALDH1A3*, *CD44*, *ITGA6*, *ZEB1*, *TCF4*), none of them unambiguously identified a "root-state" (**panel-B**), and taking the average expression over all 6 markers did not resolve this ambiguity (**panel-C**). Assuming the root-state is in the basal cluster, under Monocle-2 this state would be assigned to a subcluster representing a leaf-state (**panel A,D**), in stark contrast to LandSCENT which naturally identifies the root-state as occupying a topologically central position (see **Fig.2F**).

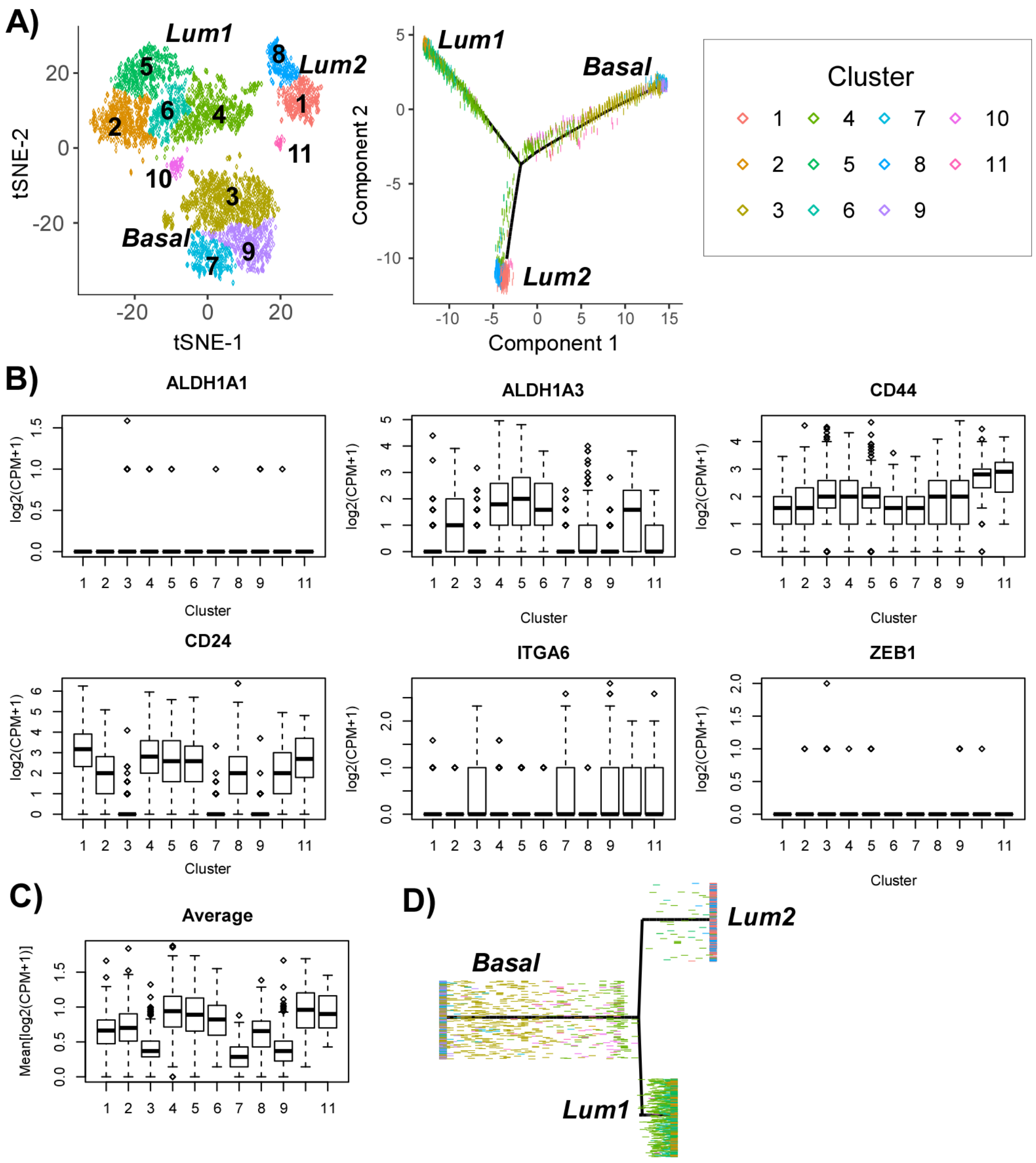

***Monocle-2 analysis. A)** Clustering (left) and lineage trajectory (right) inferred with Monocle-2 on the 10X Chromium scRNA-Seq data encompassing 3473 single cells from the mammary epithelium. **B)** Expression (log_2[counts per million+1], y-axis) for 6 putative breast stemness markers, as indicated, versus cluster-assignment from A). **C)** Average expression over the 6 stemness markers versus cluster-assignment. **D)** A typical lineage trajectory tree inferred with Monocle-2 where the root-state is in the basal group (cluster-9), failing to predict that cluster-10 is in fact the more potent state.*

SUPPLEMENTARY REFERENCES:

1. Teschendorff, A.E., Zhuang, J. & Widschwendter, M. Independent surrogate variable analysis to deconvolve confounding factors in large-scale microarray profiling studies. *Bioinformatics* **27**, 1496-505 (2011).
2. Dahlin, J.S. *et al.* A single-cell hematopoietic landscape resolves 8 lineage trajectories and defects in Kit mutant mice. *Blood* **131**, e1-e11 (2018).
3. Wolock, S.L., Lopez, R. & Klein, A.M. Scrublet: computational identification of cell doublets in single-cell transcriptomic data. *bioRxiv* (2018).
4. Pece, S. *et al.* Biological and molecular heterogeneity of breast cancers correlates with their cancer stem cell content. *Cell* **140**, 62-73 (2010).
5. Nguyen, Q.H. *et al.* Profiling human breast epithelial cells using single cell RNA sequencing identifies cell diversity. *Nat Commun* **9**, 2028 (2018).
6. Curtis, C. *et al.* The genomic and transcriptomic architecture of 2,000 breast tumours reveals novel subgroups. *Nature* **486**, 346-52 (2012).
7. Teschendorff, A.E. & Enver, T. Single-cell entropy for accurate estimation of differentiation potency from a cell's transcriptome. *Nat Commun* **8**, 15599 (2017).
8. Shi, J., Teschendorff, A.E., Chen, L. & Li, T. Quantifying Waddington's epigenetic landscape: a comparison of single-cell potency measures. *Brief Bioinform* **In Press**(2018).
9. Shehata, M. *et al.* Phenotypic and functional characterization of the luminal cell hierarchy of the mammary gland. *Breast Cancer Res* **14**, R134 (2012).
10. Qiu, X. *et al.* Reversed graph embedding resolves complex single-cell trajectories. *Nat Methods* **14**, 979-982 (2017).
